# Time Trends of Persistent Organic Pollutants in Benthic and Pelagic Indicator Fishes from Puget Sound, Washington, USA

**DOI:** 10.1007/s00244-017-0383-z

**Published:** 2017-05-20

**Authors:** James E. West, Sandra M. O’Neill, Gina M. Ylitalo

**Affiliations:** 1 Marine Resources Division, Washington Department of Fish and Wildlife, 600 Capitol Way N, Olympia, WA 98501 USA; 2 Environmental and Fisheries Sciences Division, Northwest Fisheries Science Center, National Marine Fisheries Service, National Oceanic and Atmospheric Administration, 2725 Montlake Boulevard East, Seattle, WA 98112 USA

## Abstract

We modeled temporal trends in polychlorinated biphenyls (PCBs), polybrominated diphenyl ethers (PBDEs), and dichlorodiphenyltrichloroethane and its metabolites (DDTs) in two indicator fish species representing benthic and pelagic habitats in Puget Sound, Washington, USA. English sole (*Parophrys vetulus*, benthic) index sites and larger-scale Pacific herring (*Clupea pallasii*, pelagic) foraging areas represented a wide range of possible contamination conditions, with sampling locations situated adjacent to watersheds exhibiting high, medium and low development. Consistency in analytical data throughout the study was maintained by either calculating method-bias-correction factors on paired samples as methods evolved or by analyzing older archived samples by current methods. PCBs declined moderately in two herring stocks from a low-development basin (2.3 and 4.0% annual rate of decline) and showed no change in the highly developed and moderately developed basins during a 16- to 21-year period. PCBs increased in English sole from four of ten sites (2.9–7.1%), and the remaining six exhibited no significant change. PBDEs and DDTs declined significantly in all herring stocks (4.2–8.1%), although analytical challenges warrant caution in interpreting DDT results. PBDEs declined in English sole from two high-development and one low-development site (3.7–7.2%) and remained unchanged in the remaining seven. DDTs increased in English sole from one high-development site (Tacoma City Waterway) and declined in two high-development and one low development site. As with herring, analytical challenges warrant caution in interpreting the English sole DDT results. It is likely that source controls and mitigation efforts have contributed to the declines in PBDEs and DDTs overall, whereas PCBs appear to have persisted, especially in the pelagic food web, despite bans in PCB production and use.

Although the production and use of a number of persistent organic pollutants (POPs) has been curtailed to protect environmental and human health in many countries (Tierney et al. 2014), it can be difficult to evaluate the success of these efforts over the long term, especially in aquatic environments. POPs include a wide variety of toxic industrial chemicals such as polychlorinated biphenyls (PCBs) and flame-retardant polybrominated diphenyl ethers (PBDEs), as well as chlorinated pesticides such as dichlorodiphenyltrichloroethane and its metabolites (DDTs), chlordanes, and hexachlorobenzene. These POPs can accumulate in sediments and biota where they may persist for many years (Jones and de Voogt 1999; Tierney et al. 2014). Earliest efforts to measure the level of POP contamination in marine animals (DDTs and PCBs; Jensen et al. 1969) combined with more recent time trend analyses suggest that many aquatic systems exhibited a substantial drop in DDTs and PCBs through the 1970s and 1980s [e.g., the Canadian arctic (Braune et al. 2005), the Baltic Sea (Bignert et al. 1998), and the Great Lakes of North America (Stow et al. 1994)]. Variation in biological covariates, such as fish age, lipid levels, sex, and trophic level, can confound analysis of temporal and spatial trends in biota (West et al. 2008), as well as comparisons between studies (this report). Moreover, it is difficult to maintain consistency in measuring POPs as analytical methods evolve over time.

The production and use of DDTs and PCB technical mixtures in the United States were sharply curtailed from 1972 to 1979, resulting from federal legislation banning these chemicals. In addition, Washington State passed legislation in 2008 to restrict the use of polybrominated diphenylether (PBDE) flame retardants. Washington State monitors the concentration of these three chemical groups in two marine indicator fish species representing benthic (English sole, *Parophrys vetulus*) and pelagic (Pacific herring, *Clupea pallasii*) habitats as part of a directed effort to track the health the Puget Sound ecosystem. Tissue residue recovery targets using the concept of critical POP body residues (Meador et al. 2011) are used to evaluate the success of pollution remediation efforts, on a large, ecosystem scale. The two Puget Sound fish contaminant indicators (species) reported here are part of a larger portfolio of Vital Sign indicators designed to simplify science reporting and link it to regional policy, ultimately resulting in easily communicated policy statements or targets that define the desired condition or goals (Puget Sound Partnership 2016).

Puget Sound comprises a complex, fjord-like, interconnected series of inland marine and estuarine waters in Washington State (Burns 1985; Moore et al. 2008) that exchange with the Pacific Ocean primarily via a relatively narrow waterway, the Strait of Juan de Fuca (Fig. 1). Oceanographic stratification and geographically restricted connections between deep Puget Sound basins result in recirculation of basin waters (Thomson 1994), which increases retention of regionally produced pollutants (Harrison et al. 1994). These inland marine waters drain watersheds that range from highly developed or urbanized with large cities (central Puget Sound) to moderately developed (southern Puget Sound), to rural, undeveloped lands (northern Puget Sound), resulting in marine waters that range from highly polluted to nearly pristine.

**Fig. 1:**
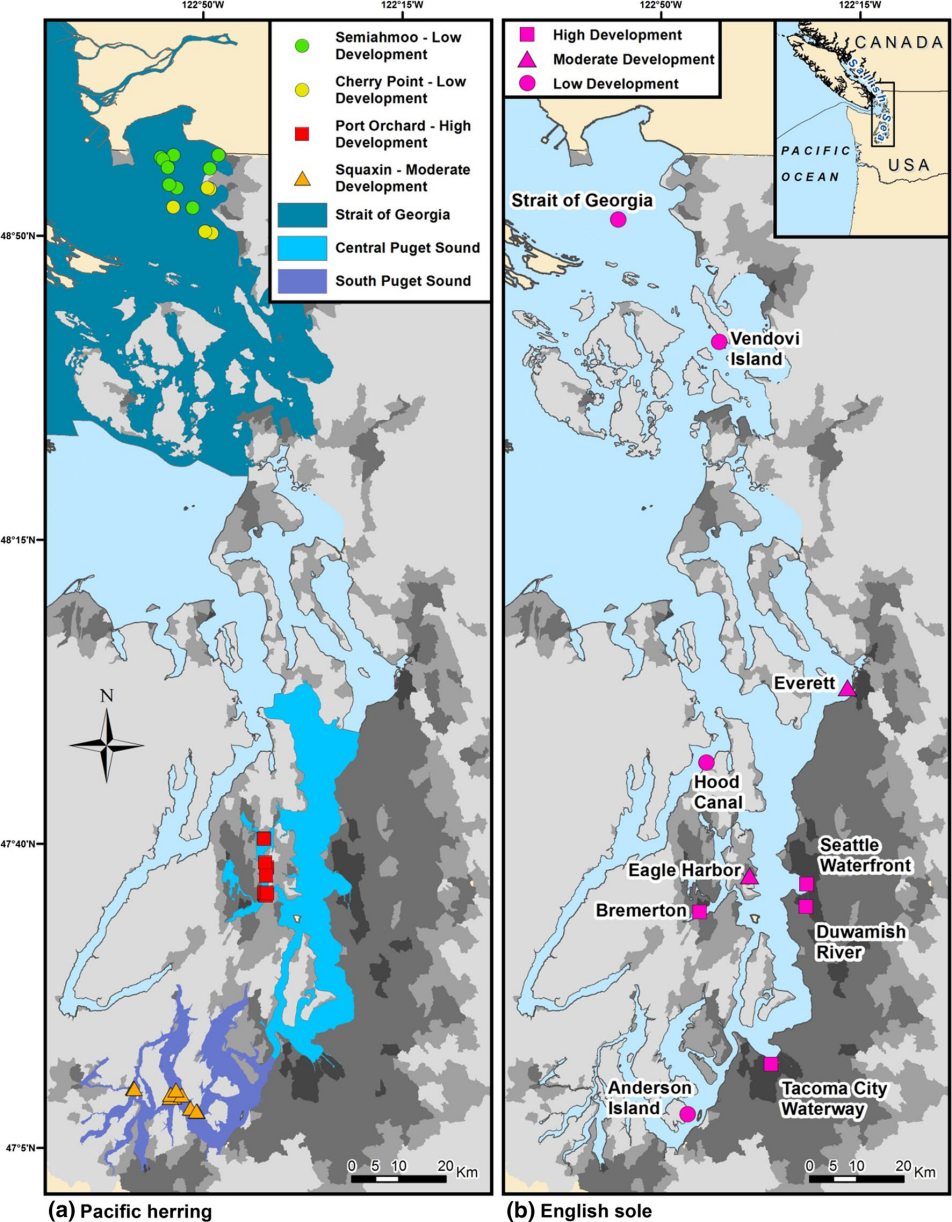
Sampling locations for Pacific herring and index sites for English sole. Pacific herring basins classified as high development, moderate development, or low development based on proximity to upland impervious surface. Impervious land-surface shown as *grey-scale* gradations from <5% (*lightest grey*) to >50% (*darkest grey*). English sole index sites classified as follows: high development (within 500 m of land with >50% impervious surface), moderate development (within 500 m of land with >25% impervious surface or within 2000 m of land with >50% impervious surface) or low development (more than 2000 m away from land with >25% impervious surface)

Previous studies have reported high levels of POPs throughout Puget Sound’s pelagic food web including its phytoplankton and zooplankton prey base (West et al. 2011a), secondary consumers, such as Pacific herring (West et al. 2008), tertiary consumers, such as Chinook salmon, *Oncorhynchus tshawytscha* (O’Neill and West 2009; Cullon et al. 2009) and gadoid codfishes (West et al. 2011b), and apex predators, including pelagic-fish-eating killer whales, *Orcinus orca* (Ross et al. 2000; Ross 2006, Ross et al. 2009; Krahn et al. 2007, 2009) and harbor seals, *Phoca vitulina* (Ross et al. 2004, 2013; Cullon et al. 2005). Based on results from these studies, POPs in these species illustrate broad, basin-wide contaminant conditions in the pelagic food web.

Ross et al. (2013) identified strongly declining trends in concentrations of PCBs from 1984 to 2010 and one other POP class (polychlorinated naphthalenes) from 1984 to 2003 in harbor seals from Southern Puget Sound. PBDEs in the same harbor seals showed a different temporal pattern, with a strong increase from 1984 to 2003 and a subsequent 40% decline in 2009. More recent POP trends in Puget Sound marine mammals remain unknown, although Hickie et al. (2007) predicted it would take between 14 and 57 years for PCB concentration to fall below an effects threshold of 17 mg total PCBs/kg blubber lipids for the populations of fish-eating killer whales that typically forage in Puget Sound.

Contamination of pelagic species with POPs has occurred at a large, basin scale in Puget Sound, whereas POP contamination of benthic and sessile species appears to be limited to urbanized areas, which is where the greatest sediment contamination occurs (Long et al. 2005). This is evident from POPs reported in blue mussels (*Mytilus trossulus*; Lanksbury et al. 2013) and English sole in Puget Sound (this report and Ylitalo et al. 1999), and consistent with other species from the California Coast (Dodder et al. 2013) and San Francisco Bay (Davis et al. 2007; Greenfield and Allen 2013).

The Washington Department of Fish and Wildlife (WDFW) has tracked POPs in indicator species representing key food-web pathways including English sole, a bottom-dwelling flatfish, as well as Pacific herring, a small, schooling mid-water planktivore, or forage fish. English sole consume infaunal invertebrates and so represent the sediment-to-biota contaminant link for contaminants that tend to accumulate in sediments. Adult Pacific herring primarily consume zooplankton and are themselves prey to virtually every large piscivorous species in Puget Sound, and so generally represent the pathway of contaminants to higher level predators including Pacific salmon, seabirds, harbor seals and killer whales.

A significant challenge for long-term toxics monitoring programs is collecting data that are comparable over long time periods either by maintaining consistent analytical methods through time, or by conducting comparison studies as methods change. This includes selection of extraction solvents and extraction methods, and subtle changes in sample handling. In addition, comparisons of contaminant levels among studies can be difficult because differing methods or summation algorithms are often used to estimate total PCBs, PBDEs, or other POP groups. Monitoring programs often rely on subsets of congeners or analytes that are readily measured using low-resolution methods, to avoid the high cost of measuring all 209 congeners using more comprehensive analytical methods such as high-resolution gas chromatography/mass spectrometry.

In the current study, we report concentrations of PCBs, PBDEs and DDTs in two primary indicator species, English sole and Pacific herring, from Puget Sound, Washington over a 16- to 21-year time period. Our objectives were (1) to apply correction factors to POP data to mitigate biases related to changes in analytical methodology and quantitation methods as techniques evolved during the monitoring periods, (2) to evaluate whether contaminant conditions are improving or worsening in Puget Sound, (3) to compare current contaminant conditions for these species for ten index sites (English sole) and three oceanographic basins (Pacific herring) representing a wide range of contamination, (4) to compare PCB summations used in our monitoring program with ∑ICES_7_, a PCB congener summation method sanctioned by the International Council for the Exploration of the Sea, and used by many European monitoring programs, (5) to compare our POP results with published critical body residues (see, Meador et al. 2011) to infer the likelihood of toxicopathic health effects on fish, and (6) to identify cases where POP body residues in fish were great enough to generate consumption advisories to protect human health.

## Methods

### Study Area and Sample Collection

Study areas for both indicator species were chosen to satisfy a wide range of needs for long-term monitoring of contaminants in Puget Sound’s pelagic and benthic food webs (Fig. 1). Three of the most abundant stocks of winter-spawning Pacific herring were selected to represent the pelagic food web in three oceanographic basins characterized by a wide range of land-development in their watersheds. The Port Orchard stock was sampled from central Puget Sound, which is characterized by highly developed or urbanized watersheds (termed high-development). The Semiahmoo stock was sampled from the southern Strait of Georgia, which is characterized by rural watersheds with little development, and a relatively direct connection to oceanic waters (termed low-development). The Squaxin stock was sampled from south Puget Sound, which exhibited relatively low land development, however its connection to oceanic waters was through the highly developed central Puget Sound, so its level of development was characterized as moderate. A fourth herring stock, Cherry Point, also occurring in the low-development southern Strait of Georgia was tracked because its unique springtime spawn-timing separates it from the other stocks, and its long history of precipitous decline has made it a priority for protection and recovery. All herring were collected in the winter (January through March) as they aggregated for spawning in predictable locations, except for Cherry Point herring, which were taken in May or June. Fish were captured using gill-nets along shores where spawning activity was occurring, or using midwater trawls in nearby off-shore waters where pre-spawners aggregated. To minimize the effects of reproduction and fish age on POP concentration, only male herring were selected, from sizes that were likely to be 3-year-old fish. Fifty male herring were sampled from each location and year. Ten composite samples, each containing five male herring were created by homogenizing whole bodies together using a food-grade stainless steel and aluminum grinder.

Ten English sole index sites were selected to represent a wide range of potential contaminant inputs for this benthic species, across the full geographic range of Puget Sound (Fig. 1). Degree of impervious surface in watershed uplands was used as a proxy for land development and also to classify index sites as follows: high development (within 500 m of land with >50% impervious surface), moderate development (within 500 m of land with >25% impervious surface or within 2000 m of land with >50% impervious surface), or low development (more than 2000 m away from land with >25% impervious surface). High development sites were typically close to shore (<500 m), whereas some moderate- and low-development sites were more than 2000 m from the shoreline used to classify them. Watershed impervious surface was estimated using the % imperviousness data layer from the 2006 National Land Cover Database (Fry et al. 2011; Wickham et al. 2013) for closest catchment areas draining into Puget Sound.

Although English sole are known to make long-distance movements for spawning in winter months before homing back to their feeding ground in the spring and summer months (Moser et al. 2013), they are typically sedentary during spring and summer months (Day 1976), where they exhibit small, predictable foraging ranges (O’Neill et al. 2007). Because they are thought to feed little during the winter (Day 1976), their POP tissue burdens likely represent local conditions of their spring/summer habitats. All English sole were captured using a bottom trawl, and skin-off muscle tissue was later resected from either fresh/iced or frozen/thawed specimens. An equal mass of muscle (typically 10 g) was resected from each of 60–120 specimens per site per year and combined randomly to create three or six composite groups of 10–20 fish each. Composited tissues were homogenized using hand-held stainless steel electric mixers.

### Chemical Analyses

Two analytical methods, two extraction methods, and two extraction solvents were used to measure POPs in English sole and Pacific herring samples over the duration of this study. The most recently collected samples were analyzed by gas chromatography/mass spectrometry (GC/MS) and accelerated solvent extraction (ASEx) with methylene chloride (MeCl) according to Sloan et al. (2014). Specifications for instruments, supplies and materials used in all GC/MS runs are detailed in Sloan et al. (2004, 2014). Earlier collected samples were analyzed via high-performance liquid chromatography with photodiode array (HPLC/PDA) detection according to Krahn et al. (1994). The HPLC/PDA analytical method was conducted with either manual solvent extraction (MSEx) and equal volumes of pentane and hexane (PenHex), or ASEx using MeCl.

Tissue samples were analyzed using GC/MS with ASEx according to Sloan et al. (2014) from 2005 to 2015 for PCBs and DDTs and from 1994 to 2015 for PBDEs (using a combination of real-time and archived samples). In brief, this method comprised three steps: (1) ASEx of tissue using MeCl, (2) two-step cleanup of the MeCl extract by silica/aluminum columns and size-exclusion high-performance liquid chromatography (SEC HPLC) to remove lipids and other biogenic compounds, and (3) quantitation of POPs using GC/MS with selected-ion-monitoring (SIM). The ASEx provided an extract that was used for analyte recovery and gravimetric lipid evaluation. Accuracy of the instrument was improved by including chemical ionization filaments (used to increase source temperature), a cool on-column injection system in the GC, and a guard column before the analytical column. Point-to-point calibration was used improve data fit over the full range of GC/MS calibration standards (Sloan et al. 2004). Forty-seven PCBs, 11 PBDEs, and 5 DDTs were routinely detected in all samples.

Total PCBs analyzed by GC/MS were estimated using a simple algorithm based on a subset of 17 commonly detected PCB congeners representing homologs containing three to ten chlorine atoms (IUPAC numbers 18, 28, 44, 52, 66, 101, 105, 118, 128, 138, 153, 170, 180, 187, 195, 206, and 209) wherein the sum of detected values for these 17 congeners was multiplied by 2 to estimate total PCBs. This algorithm was developed in the context of a US nationwide toxics monitoring program from empirical data (Lauenstein and Cantillo 1993), as a cost-effective way to estimate total PCBs from a small subset of congeners using relatively inexpensive low-resolution analytical techniques. Total PCBs were estimated by Lauenstein and Cantillo (1993) using 18 congeners (those above, plus PCB 8); however, PCB 8 was not quantitated in the current study, so the algorithm employed herein summed only 17 congeners, with the estimation of total PCBs hereafter referred to as 2∑_17_PCBs. Although PCB 8 was detected in 30 samples of fish tissue in another study of pelagic Puget Sound fish using high-resolution GC/MS methods (West et al. 2011b), concentrations ranged only from 0.0014 to 0.012 ng/g wet wt, suggesting that it would be undetectable by the methods used in this study. Lauenstein and Cantillo’s (1993) 2∑_18_PCBs provided excellent agreement with total PCBs estimated from a total chlorination method in their study, and 2∑_17_PCBs in the current study agreed well with total PCBs estimated from the sum of 209 congeners measured by high resolution methods (linear regression of ∑209 congeners by 2∑_17_PCBs, *r* ^2^ = 0.997, *p* = 0.003, slope coefficient =0.991, intercept not significant, *p* = 0.892) for a subset of five English sole and herring from this study.

To facilitate broader interpretation of our 2∑_17_PCBs results, we also compared them with ∑ICES_7_ PCBs, a commonly calculated summed PCB estimate in European biota (International Council for the Exploration of the Sea 1987). In a linear regression of 1089 fish and invertebrate samples analyzed by WDFW from 1994 to 2014, the 2∑_17_PCBs were approximately 2.6 times higher than ∑ICES_7_ PCBs calculated from the same samples (linear regression, *r* ^2^ = 0.998, *p* < 0.0001, slope coefficient = 2.58, intercept = 0.61).

Summed PBDEs were calculated as the sum of detected values for the 11 identified PBDE congeners (IUPAC numbers 28, 47, 49, 66, 85, 99, 100, 153, 154, 155, 183), hereafter referred to as ∑_11_PBDEs, and summed DDTs were calculated as the sum of detected values for five identified DDT compounds (*o*,*p’*-DDD, *p*,*p’*-DDD, *p*,*p’*-DDE, *o*,*p’*-DDT, and *p*,*p’*-DDT), hereafter referred to as ∑_5_DDTs. In cases where no single PBDE or DDT analyte was detected in a sample for its group, a value of the greatest LOQ for all analytes in a group was substituted for the total. This value was typically less than 1 ng/g wet wt. At least one PCB congener was detected in every sample.

Although all PBDEs were analyzed by the most current method by combining archived tissues and real-time samples, before 2005 PCBs and DDTs were analyzed using HPLC/PDA detection according to Krahn et al. (1994) (see correction procedure in “Method Performance” section). In addition the method of extraction changed from MSEx using PenHex from 1997 to 2003 (Sloan et al. 2005) to ASEx using MeCl (2003–2005). Briefly, for the HPLC/PDA, sample extracts were reduced in volume to approximately 1 ml and the POPs were separated from interfering compounds (e.g., lipids and aromatic compounds) on a gravity flow cleanup column that contained neutral, basic, and acidic silica gels eluted with PenHex (1997–2003) or MeCl (2003–2005). PCB congeners (IUPAC numbers 77, 105, 101, 110, 118, 126, 128, 138, 153, 156, 157, 169, 170/194, 180, and 189) were resolved from five chlorinated pesticides (*o*,*p’*-DDD, *p*,*p’*-DDD, *p*,*p’*-DDE, *o*,*p’*-DDT, *p*,*p’*-DDT) by HPLC on two Cosmosil PYE analytical columns, connected in series and cooled to 16 °C. Compounds were measured by an ultraviolet (UV) PDA and were identified by comparing their UV spectra (200-310 nm) and retention times to those of reference standards in a library. Analyte purity was confirmed by comparing spectra within a peak to the apex spectrum. The concentration of total PCBs analyzed by HPLC/PDA was estimated as the sum of the concentrations of the 16 PCBs listed above (based on individual response factors) plus the sum of the concentrations of other unidentified PCBs calculated by summing areas of peaks identified as PCBs and using an average PCB response factor. Summed DDTs were calculated as the sum of detected values of the five DDT isomers. If no DDT isomer was detected in a sample, the greatest LOQ for the five isomers (typically <0.5 ng/g, wet wt) was substituted as the summed DDT value for that sample.

Lipid concentration was determined gravimetrically in all samples from all years. For PCBs and DDTs from 1997 to 2003, lipids were extracted using MSEx with PenHex and homogenized with a tissue grinder (Sloan et al. 2004). Lipids for PCBs and DDTs from 2003 onward and for all PBDEs (1994–2015) were extracted using ASEx with MeCl (Sloan et al. 2014).

### Method Performance

Recoveries of analytes from the National Institute of Standards and Technology (NIST) Standard Reference Materials (SRMs) were considered the primary measure of analytical performance for all methods, especially over the long-term, as equipment and supplies can vary through time, even for standardized methods. Additional quality control measures, including sample replication and addition of surrogate standards, were used to identify problematic batches according to Sloan et al. (2004, 2014); however, these metrics typically passed quality control limits and so are not further reported here. A sample of NIST SRM 1974a (blue mussel) was analyzed with each HPLC/PDA sample batch; NIST SRM 1947 (Lake Michigan fish tissue) samples were run with GC/MS batches before 2015 and NIST SRM 1946 (Lake Superior fish tissue) was used after 2015. All batches contained 12–14 field samples. A NIST SRM quality control limit was used to flag poorly analyzed batches for reanalysis; batches were rejected if ≥30% of individual analytes measured in the NIST SRM exceeded 30% of the 95% confidence interval range of the published NIST certified concentrations and reanalyzed until they complied with recovery controls.

SRM recovery target concentrations were further calculated for each of the three POP classes by summing the NIST certified or reference values for analytes that were analyzed in field samples. Measuring recovery of SRM analytes through time provided an estimate of variance for method performance, which we calculated as the 95% confidence interval (CI) for the year factor in a linear regression of the SRM analyte recovery by year (or the CI of the mean analyte % recovery if the year coefficient was not significant). Significant year coefficients from field sample regressions falling within the 95% confidence interval from SRM % recovery regressions were deemed indistinguishable from method variability and censored as not significant as described below.

For the 17 congeners used in 2∑_17_PCB measured by GC/MS, certified values for PCB 66 and 195 were unavailable from NIST, so % recovery was calculated using a sum of the other 15 congeners from the 2∑_17_PCB list. Ninety-six percent of the resulting ∑_15_PCB concentrations recovered from SRM 1947 and SRM 1946 were within 15% of the NIST certified 95% confidence interval, indicating a high degree of accuracy. ∑_15_PCB recoveries in SRMs from the GC/MS showed no significant time-related bias, based on a linear regression of SRM % recovery by year for English sole (*p* = 0.49) and herring (*p* = 0.68). Overall, the 95% confidence interval for the mean ∑_15_PCB SRM recovery was only ±2.1% for English sole and ±1.9% for herring; accounting for this variability in the % recovery of the SRM, significant time trend year coefficients (i.e., slopes) from field sample regressions of 2∑_17_PCBs by year within these ranges were considered indistinguishable from analytical variability and subsequently censored as not significant. Eighty-six percent of summed PBDE SRM recoveries were within 15% of the NIST-certified 95% confidence interval. PBDE SRM recoveries did not vary significantly with time for English sole and only weakly for herring batches (linear regression of PBDE recovery by time, *p* = 0.057, adjusted *r* ^2^ = 0.084 for English sole; *p* = 0.012, adjusted *r* ^2^ = 0.22 for herring) with slight annual rates of decline of 0.86 and 0.83%. PBDE time trends in field samples falling within the 95% confidence interval of the PBDE SRM % recovery regression slope (−1.7 to −0.024% for English sole and −1.4 to −0.24% for herring) were considered indistinguishable from analytical variability, and subsequently censored as not significant. Eighty-nine percent of summed DDT SRM recoveries were within 15% of the NIST-certified 95% confidence interval; however, SRM recoveries of DDTs decreased consistently through time (linear regression of summed DDT recovery by year, *p* < 0.001, adjusted *r* ^2^ = 0.52 for English sole; *p* < 0.001, adjusted *r* ^2^ = 0.67 for herring). DDT time trends reported herein for field collected samples were considered indistinguishable from this bias if they fell within the 95% confidence interval of the slope coefficient from the SRM time regressions (−2.6 to −1.3% for English sole; −2.9 to −1.6%) and subsequently censored as not significant.

SRM recoveries for PCBs analyzed by HPLC/PDA were lower than the GC/MS method; 16% of PCBs, calculated as a sum of 8 congeners in common between the field samples and SRM certified values, were within 15% of the NIST-certified 95% confidence interval. Recoveries of DDTs from SRMs analyzed by the HPLC/PDA were hampered by the presence of unidentified interfering compounds, and so SRM recoveries were unusable for that analyte group. PBDEs were not measured by the HPLC/PDA method. However, DDT and PCB results from HPLC/PDA analyzed field samples were corrected using a robust sample-to-sample comparison described below. This method corrected HPLC/PDA results to GC/MS standards, essentially precluding the need to evaluate or correct for SRM recoveries from the HPLC/PDA results. Moreover, the SRM recoveries for PCBs and DDTs appeared to be consistent through time for each species, based on a visual inspection of the SRM recoveries; low sample size prevented computing valid regression models for the HPLC/PDA methods.

### Method-Bias Corrections for Time Trends

To correct for bias in estimated total PCB estimates associated with analytical method changes, 76 samples that had been analyzed by the older methods were reanalyzed using the GC/MS with ASEx described above: 14 samples by the HPLC/PDA method with MSEx and 62 samples by the HPLC/PDA with ASEx. Correction factors were calculated by regressing 2∑_17_PCBs from the GC/MS with ASEx method against estimated total PCBs calculated from each of the HPLC/PDA groups in the same samples. In both cases, the HPLC/PDA methods underestimated 2∑_17_PCBs; by approximately 33% for the MSEx group (linear regression of 2∑_17_PCBs by total PCBs, *r* ^2^ = 0.90, *p* < 0.001, slope coefficient = 1.331, intercept not significant, *p* = 0.97), and 27% for the ASEx group (*r* ^2^ = 0.94, *p* < 0.001, slope coefficient = 1.267, intercept not significant, *p* = 0.83).

The same five DDT analytes were measured across both analytical and extraction methods, precluding a need for method-corrections based on summation of differing chemicals in the group. However, a comparison of ∑_5_DDTs across the methods revealed a difference in quantitation of individual DDTs related to the solvent extraction method. ∑_5_DDTs measured by the HPLC/PDA method with ASEx were similar to results from the GC/MS with ASEx and therefore required no adjustment (linear regression of paired ∑_5_DDTs by both methods; *r* ^2^ = 0.95, *p* < 0.001, slope coefficient = 0.95, intercept not significant, *p* = 0.77); however, ∑_5_DDTs measured by HPLC/PDA with MSEx were approximately 45% lower than the GC/MS method (linear regression; *r* ^2^ = 0.95, *p* < 0.001, slope coefficient = 1.45, intercept not significant, *p* = 0.68). Hence, ∑_5_DDTs were adjusted upwards according to this latter regression model, for samples that were only analyzed by the HPLC/PDA and MSEx.

Beginning in 2005, PBDEs were always measured in both species of fish by the GC/MS method described above with ASEx, and all PBDE in prior years were determined from archived tissue samples. Thus, no adjustments were made for any PBDE data.

### Data Analysis

For evaluation of time trends, we used multiple linear regressions of 2∑_17_PCBs, ∑_11_PBDEs, and ∑_5_DDTs over time, with year as the independent variable, to evaluate whether observed time trends in POPs were statistically significant. The contribution of biological covariates in explaining POP variability also was evaluated. Composite-mean fish length (MCL) was used as a proxy for fish age, which can be considered an estimate of total potential exposure time to contamination. Because the POPs measured in this study are considered lipophilic, total nonvolatile extractibles (percent lipids) were included to help control for variability in that measure. The sex ratio (proportion of male fish) was included to help control for potential differences related to loss of POPs in reproducing female fish. Automatic stepwise regression models were run using SYSTAT (2007), for each log-transformed POP total in separate runs, with the covariates listed above and their interactions. Although some interaction terms were occasionally significant, all interaction terms explained little POP variation (usually less than 3%), and including them did not appreciably change results. Hence, for simplicity they were omitted from the final regression models. Geometric mean POP concentrations and confidence intervals were adjusted for significant covariate terms by predicting the POP concentration through time from the multiple linear regression models, using the grand mean value for MCL, lipids, or sex ratio, when they were included in the final model. Partial coefficients of determination (*r* ^2^) were calculated at each step of the forward stepwise multiple linear regression to estimate the contribution of each significant covariate in explaining POP concentration in the final model. A significant (*p* > 0.05) year coefficient in the final model was accepted as evidence for a significant time trend; however, as an additional guard against overinterpreting weak trends, year coefficients with *r* ^2^ < 0.10 (i.e., time explaining <10% of POP variability) were censored as trivial (and models presented as dashed lines in graphs).

To evaluate the most current status of POPs in English sole and herring, we used analysis of variance (ANOVA) to test the significance of observed log-transformed POP differences between the ten English sole index sites collected in 2015 and between the four herring stocks collected in 2014. Although lipids, fish size, and sex ratio were tested as ANOVA covariates, none was a significant contributor in explaining POP variation, so POPs were not adjusted by these factors for the current status comparison.

## Results

Time series data for herring comprised 12 sampling years spanning a 16-year period from 1999 to 2014 for two stocks (Port Orchard and Squaxin), 9 years over the same period for Semiahmoo and 6 years of sampling over a 21-year period for Cherry Point (Table 1). The time series for English sole covered 9 to 12 sampling years over an 18- or 19-year span for all sites except the Duwamish River, which was sampled in 1997 and then not again until 2007–2015 (Table 1). The most recent years for both species were analyzed below to represent current status.

**Table 1:** Arithmetic mean concentration (ng/g in wet weight and lipid weight) of PCBs, PBDEs, and DDTs and biological metrics for composite samples of Pacific herring (*Clupea pallasii*; *n* = 10 fish per composite) and English sole (*Parophrys vetulus*; *n* = 10–20 fish per composite) for all years sampled. Contaminant means not corrected for biological covariates. Herring represented by four stocks in three oceanographic basins and sole from ten index sites. na indicates not applicable; nfs indicates no fish sampled. Mean composite length (MCL) is the mean standard length in mm of all fish in each sample

	Sex ratio (% males)	MCL (mm)	Lipids (%)	2∑_17_PCBs		ICES_7_ PCBs		∑_11_PBDEs		∑_5_DDTs	
				Wet	Lipid	Wet	Lipid	Wet	Lipid	Wet	Lipid
*Cherry Point herring*											
1994	na	162	3.1	89	2900	34	1100	30	980	19	600
1999	na	176	3.3	58	1800	22	680	nfs	nfs	19	530
2001	na	172	2.7	61	2300	23	900	24	900	18	630
2007	na	171	2.5	56	2400	21	900	23	970	12	480
2012	na	171	4.2	50	1200	19	480	17	400	7.1	170
2014	na	159	3.0	46	1500	18	580	13	410	6.7	220
*Port Orchard herring*											
1999	na	170	6.5	200	3100	77	1200	nfs	nfs	36	500
2000	na	169	5.1	230	4700	87	1800	nfs	nfs	34	620
2001	na	169	5.6	230	4200	90	1600	96	1600	27	440
2002	na	169	5.2	240	4700	91	1800	70	1500	25	410
2003	na	172	5.6	250	4700	96	1800	96	2100	23	350
2004	na	166	5.5	160	3000	63	1200	48	910	16	250
2006	na	171	8.3	210	2600	81	1000	47	580	18	230
2007	na	164	6.7	220	3300	83	1300	59	900	19	300
2008	na	167	4.8	180	3800	68	1500	39	890	16	350
2010	na	157	4.9	160	3400	62	1300	25	530	13	280
2012	na	181	5.6	220	4100	86	1600	53	960	19	350
2014	na	164	5.5	190	3400	72	1300	30	550	12	220
*Semiahmoo herring*											
1999	na	175	5.1	58	1100	22	430	nfs	nfs	22	400
2000	na	168	4.9	55	1200	21	450	nfs	nfs	23	440
2001	na	171	3.8	53	1500	20	570	29	850	19	460
2002	na	166	4.2	38	940	15	360	19	470	17	350
2003	na	171	4.8	43	920	16	350	25	580	14	240
2004	na	171	3.5	39	1100	15	440	17	520	15	430
2006	na	158	4.8	31	660	12	250	9.3	200	11	230
2012	na	176	4.2	44	1100	17	420	18	420	12	280
2014	na	162	3.2	18	550	6.8	210	5.5	170	5.5	180
*Squaxin herring*											
1999	na	165	9.5	240	2800	91	1100	nfs	nfs	38	400
2000	na	157	7.7	170	2200	65	850	nfs	nfs	25	290
2001	na	156	6.8	170	2600	67	990	56	890	23	300
2002	na	163	6.1	280	4600	110	1800	88	1500	37	520
2003	na	166	6.8	240	3700	93	1400	120	1800	29	370
2004	na	160	6.3	170	2700	64	1000	71	1200	19	250
2006	na	160	10	180	1800	70	690	58	580	18	170
2007	na	162	8.9	170	1900	64	720	45	500	17	200
2008	na	153	6.7	160	2500	63	960	41	620	17	260
2010	na	143	5.2	130	2400	48	940	29	560	13	240
2012	na	166	8.5	250	2900	94	1100	60	710	23	270
2014	na	163	4.4	210	4800	81	1800	44	1000	17	410
*Tacoma City Waterway sole*											
1997	43	300	0.27	22	8400	8.5	3200	nfs	nfs	0.9	420
1998	38	278	0.36	32	9300	12	3600	nfs	nfs	0.6	190
1999	55	255	0.41	60	15,000	23	5700	nfs	nfs	4.1	1100
2000	50	278	0.49	47	9600	18	3700	nfs	nfs	2.9	620
2001	55	279	0.59	98	17,000	38	6400	nfs	nfs	9.0	1600
2003	80	263	0.58	200	35,000	77	13,000	nfs	nfs	8.6	1500
2005	61	261	0.40	96	24,000	37	9300	12	3000	6.5	1600
2007	48	258	0.23	52	23,000	20	9000	3.2	1400	2.1	940
2009	63	263	0.16	110	69,000	44	27,000	7.7	4700	5.2	3100
2011	50	278	0.27	110	40,000	41	15,000	9.3	3400	3.6	1300
2013	59	253	0.39	95	25,000	36	9700	7.5	2100	3.7	990
2015	54	261	0.28	99	35,000	38	14,000	6.0	2200	3.1	1100
*Duwamish River sole*											
1997	58	285	0.35	123	35,000	47	13,000	nfs	nfs	4.2	1300
2007	57	274	0.50	290	57,000	110	22,000	3.9	780	5.9	1200
2009	48	268	0.23	310	141,000	120	54,000	4.3	2000	6.1	2700
2011	36	260	0.44	270	62,000	110	24,000	6.4	1500	4.9	1100
2013	44	270	0.43	280	65,000	110	25,000	6.9	1600	4.7	1100
2015	47	274	0.34	270	81,000	100	31,000	4.3	1300	4.0	1200
*Eagle Harbor sole*											
1998	43	283	0.36	22	6100	8.5	2400	nfs	nfs	0.2	75
1999	30	283	0.37	27	7300	10	2800	nfs	nfs	1.9	580
2000	35	287	0.51	28	5500	10	2100	nfs	nfs	1.6	330
2001	60	279	0.52	33	6300	12	2400	nfs	nfs	2.3	460
2007	36	277	0.43	28	6400	10	2500	1.2	270	0.9	200
2009	22	275	0.40	33	8300	12	3200	0.63	160	0.9	210
2011	35	272	0.28	78	27,000	30	11,000	5.0	1700	1.8	630
2013	26	279	0.42	47	11,000	18	4400	1.8	450	1.1	260
2015	26	290	0.37	29	8100	11	3100	1.0	290	0.6	180
*Seattle Waterfront sole*											
1997	55	290	0.46	70	14,000	27	5200	nfs	nfs	3.4	710
1998	57	259	0.34	59	18,000	23	6800	nfs	nfs	0.3	87
1999	73	275	0.52	36	7300	14	2800	nfs	nfs	2.6	530
2000	52	307	0.66	26	3900	10	1500	nfs	nfs	2.2	330
2001	83	285	0.67	57	9000	22	3400	nfs	nfs	3.5	570
2003	73	293	0.88	89	10,000	34	4000	nfs	nfs	4.3	490
2005	87	280	0.69	45	6500	17	2500	5.8	850	2.0	350
2007	92	261	0.35	92	27,000	35	10,000	7.5	2200	2.9	830
2009	88	258	0.43	50	12,000	19	4600	3.4	830	1.5	370
2011	82	266	0.45	100	35,000	40	14,000	6.8	2100	2.4	770
2013	86	269	0.80	77	9900	30	3800	3.2	400	1.7	220
2015	63	271	0.53	160	31,000	61	12,000	4.5	910	3.0	600
*Hood Canal sole*											
1997	22	287	0.32	2.2	690	0.9	260	nfs	nfs	0.3	120
1998	12	294	0.57	2.0	340	0.8	130	0.70	120	0.6	120
1999	32	274	0.21	3.2	1600	1.2	600	nfs	nfs	0.7	540
2000	12	276	0.45	3.2	710	1.2	270	nfs	nfs	0.4	110
2001	30	291	0.73	6.7	970	2.6	370	nfs	nfs	1.0	140
2003	58	279	0.65	14	2200	5.3	840	1.1	170	1.7	290
2005	40	277	0.41	5.3	1300	2.0	510	0.54	130	0.6	160
2007	31	275	0.40	4.5	1300	1.7	480	0.68	160	0.5	140
2009	38	296	0.54	4.6	890	1.8	340	0.51	100	0.4	72
2011	38	262	0.43	4.2	980	1.6	380	0.36	86	0.3	60
2013	63	274	0.43	7.2	1700	2.8	660	0.64	150	0.6	150
2015	42	268	0.39	9.8	2400	3.8	940	0.46	120	0.5	130
*Anderson Island sole*											
1997	40	272	0.33	7.8	2400	3.0	930	nfs	nfs	0.7	260
1998	32	268	0.35	9.8	3000	3.8	1100	nfs	nfs	0.4	120
1999	25	265	0.27	9.3	3400	3.6	1300	nfs	nfs	1.1	510
2000	15	254	0.45	9.6	2200	3.7	830	nfs	nfs	0.7	160
2001	15	282	0.50	13	2600	5.0	1000	nfs	nfs	1.2	250
2003	10	268	0.51	24	4900	9.3	1900	nfs	nfs	1.4	300
2005	14	276	0.32	16	4900	6.1	1900	1.5	450	0.8	250
2007	5	280	0.33	12	3700	4.5	1400	0.66	210	0.5	150
2009	8	272	0.22	15	7300	5.9	2800	1.0	490	0.7	320
2011	10	263	0.39	13	3400	5.0	1300	2.0	510	0.4	110
2013	8	267	0.37	19	5000	7.1	1900	2.1	570	0.7	190
2015	5	262	0.32	20	6900	7.8	2700	1.3	460	0.6	220
*Everett sole*											
1997	28	291	0.24	15	5500	3.8	2100	nfs	nfs	2.0	900
1998	28	253	0.28	5.1	1800	1.9	690	nfs	nfs	0.6	290
1999	28	264	0.29	7.9	3100	3.0	1200	nfs	nfs	0.6	350
2000	38	275	0.44	9.3	2100	3.6	810	nfs	nfs	0.9	210
2001	18	278	0.51	23	4600	8.9	1800	nfs	nfs	2.2	460
2003	18	274	0.58	18	3200	7.1	1200	nfs	nfs	1.0	180
2005	19	290	0.40	21	5200	8.0	2000	1.6	380	1.0	260
2007	26	270	0.43	22	5000	8.3	1900	1.9	430	0.7	160
2009	18	273	0.27	17	6500	6.7	2500	0.86	320	0.6	240
2011	25	274	0.31	32	10,000	12	3900	3.9	1300	1.0	320
2013	14	275	0.35	22	6400	8.6	2400	2.3	660	0.7	210
2015	20	276	0.34	24	7100	9.0	2700	1.6	470	0.7	200
*Bremerton sole*											
1997	35	350	0.38	57	16,000	21	6000	nfs	nfs	1.6	530
1998	32	327	0.46	130	28,000	51	11,000	4.8	1000	2.6	590
1999	40	320	0.46	75	17,000	29	6400	nfs	nfs	1.8	410
2000	15	343	0.63	87	14,000	34	5300	nfs	nfs	2.1	330
2001	52	306	0.54	160	32,000	63	12,000	nfs	nfs	4.2	860
2003	38	325	0.60	170	28,000	65	11,000	6.5	1100	3.5	620
2005	22	319	0.53	75	14,000	29	5400	1.8	330	1.6	300
2007	23	306	0.65	76	12,000	29	4500	1.4	220	1.2	190
2009	26	311	0.40	59	15,000	23	5700	1.3	340	1.3	340
2011	13	354	0.32	130	42,000	49	16,000	4.5	1500	1.8	600
2013	27	327	0.49	70	14,000	27	5500	1.8	370	1.1	220
2015	26	314	0.49	58	13,000	22	5100	1.7	370	1.3	300
*Str. Georgia sole*											
1997	3	355	0.28	2.2	820	0.9	310	nfs	nfs	1.0	450
1998	2	351	0.25	4.2	1700	1.6	640	nfs	nfs	0.4	220
1999	5	380	0.24	2.1	900	0.8	350	nfs	nfs	0.8	440
2000	12	350	0.81	4.9	610	1.9	240	nfs	nfs	1.1	130
2001	5	348	0.63	5.5	890	2.1	340	nfs	nfs	2.0	330
2003	26	306	0.40	6.8	1800	2.6	680	nfs	nfs	1.6	480
2005	15	325	0.42	1.2	270	0.4	110	0.83	210	0.5	120
2007	13	299	0.45	1.4	330	0.5	130	0.97	240	0.6	140
2009	9	321	0.31	2.7	920	1.1	350	0.50	170	0.5	150
2011	13	328	0.37	6.0	1900	2.3	720	1.4	410	0.9	270
2013	1	375	0.49	1.2	240	0.4	94	0.31	63	0.5	96
2015	2	372	0.49	4.9	1000	1.9	400	0.28	60	0.5	98
*Vendovi Is. sole*											
1997	8	255	0.22	1.0	450	0.4	170	nfs	nfs	0.5	300
1998	23	275	0.29	2.5	880	1.0	340	nfs	nfs	0.3	110
1999	3	278	0.41	2.0	500	0.8	190	nfs	nfs	0.6	160
2000	5	279	0.45	2.3	520	0.9	200	nfs	nfs	0.7	180
2001	10	275	0.45	3.2	730	1.2	280	nfs	nfs	0.9	210
2003	10	286	0.48	5.2	1100	2.0	430	nfs	nfs	1.2	260
2005	4	299	0.42	0.9	210	0.3	82	0.99	240	0.5	110
2007	7	285	0.28	1.6	600	0.6	230	0.81	290	0.6	220
2009	13	284	0.36	2.0	540	0.8	210	0.33	95	0.5	140
2011	5	282	0.28	5.4	1900	2.1	740	1.3	450	0.6	220
2013	6	299	0.38	1.8	470	0.7	180	0.61	160	0.6	150
2015	8	297	0.34	4.2	1400	1.6	530	0.41	160	0.3	110

**Fig. 2:**
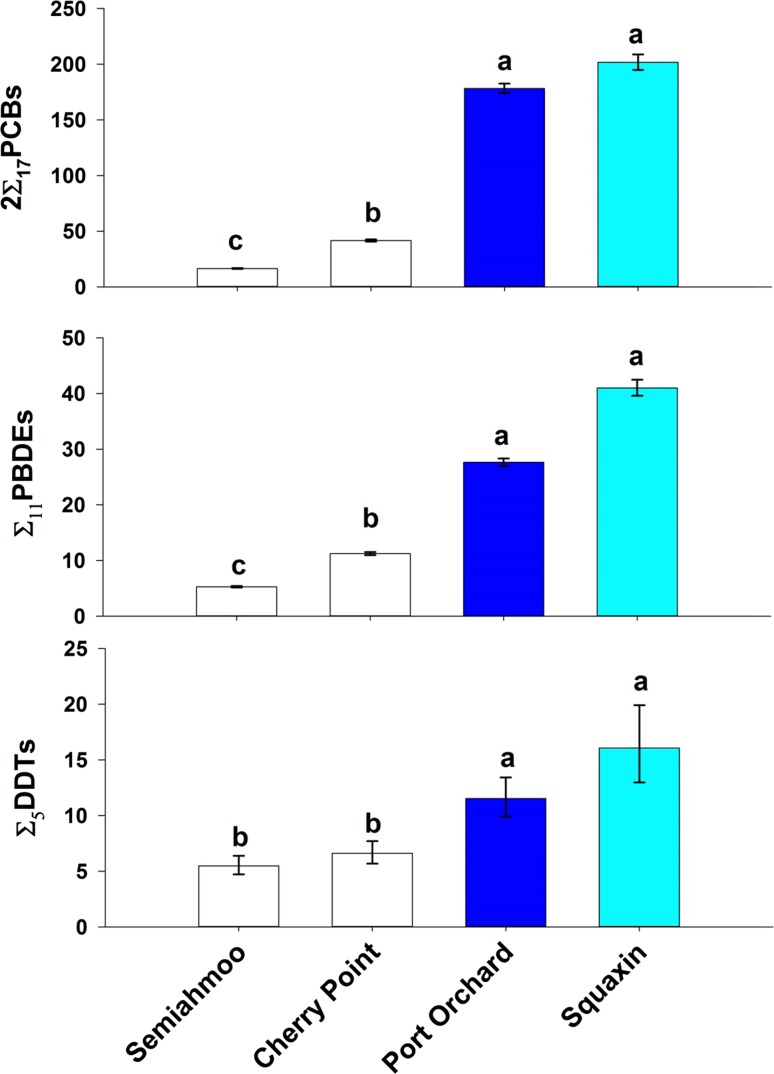
Geometric mean concentration of 2∑_17_PCBs, ∑_11_PBDE, and ∑_5_DDTs (ng/g wet wt) in four stocks of Pacific herring from 2014, the most recent sampling period. The levels of basin development are illustrated by *color*; *white* for low, *cyan* for moderate and *dark blue* for high. For each POP class, sites with the *same lower case letter* were not significantly different from each other

**Fig. 3:**
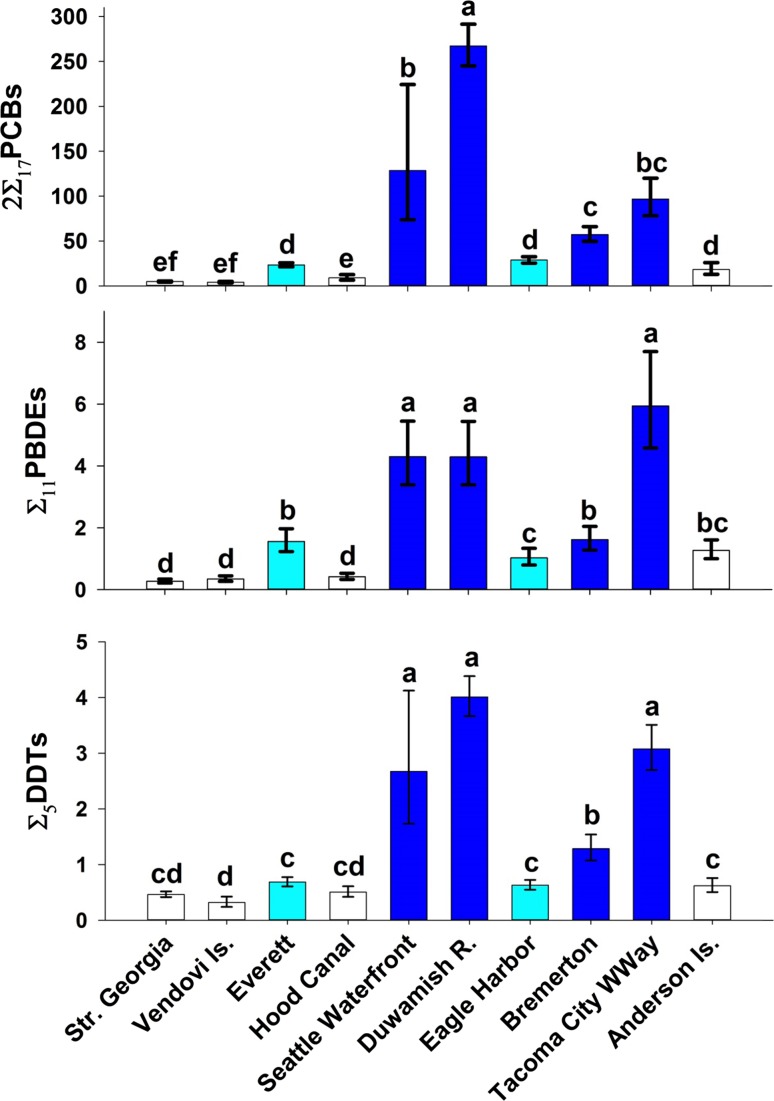
Geometric mean concentration of 2∑_17_PCBs, ∑_11_PBDE, and ∑_5_DDTs (ng/g wet wt) in English sole from ten index sites in 2015, the most recent sampling effort. Levels of site development are illustrated by color; *white* for low, *cyan* for moderate and *dark blue* for high. For each POP class, sites with the *same lower case letter* were not significantly different from each other

Of the three POP classes reported in our study, 2∑_17_PCBs dominated in concentration for both fish species, followed by ∑_11_PBDE and ∑_5_DDTs. The 2∑_17_PCB concentrations from the most current sampling were 3–6 times greater than ∑_11_PBDE and 3–16 times greater than ∑_5_DDTs in 2014 Pacific herring (Fig. 2) and 10–63 times greater than ∑_11_PBDE and 10–67 times greater than ∑_5_DDTs in 2015 English sole (Fig. 3). Percent lipids in whole body herring ranged from 2.5 to 10%, with highest values determined in herring from Squaxin (4.4–10%) and lowest in those from Cherry Point (2.5–4.2%). Size of the male herring ranged relatively narrowly (by study design) from 143 to 181 mm SL (Table 1). Lipid values for English sole muscle were consistently low across all years and all sites, ranging from 0.16 to 0.88% (Table 1). Size of English sole (as mean composite length) ranged from 253 to 380 mm TL, and the proportion of male fish (sex ratio) in the composites ranged widely from 1 to 92%.

### Pacific Herring Current Status

PCB concentrations in male Pacific herring in 2014 were highest in fish from the Squaxin stock (210 ng/g, moderately developed basin) and Port Orchard stock (190 ng/g, highly developed basin), followed by 46 ng/g in fish from Cherry Point and 18 ng/g from Semiahmoo stocks, both from a basin characterized by low-development (Table 1; Fig. 2). Levels of ∑_11_PBDEs and ∑_5_DDTs showed a similar pattern, with decreasing concentrations from Squaxin to Port Orchard to Cherry Point to Semiahmoo. Fish length and lipid covariates (or their interactions) were sometimes significant in the ANCOVA models comparing locations in the current status analysis; however, they were dropped from the final model, because they consistently accounted for a minor amount (<3%) to the variability in concentration in any of the POPs. Geometric means and confidence intervals (shown in Fig. 2) were not adjusted for any covariates.

### Pacific Herring Time Trends

**Fig. 4:**
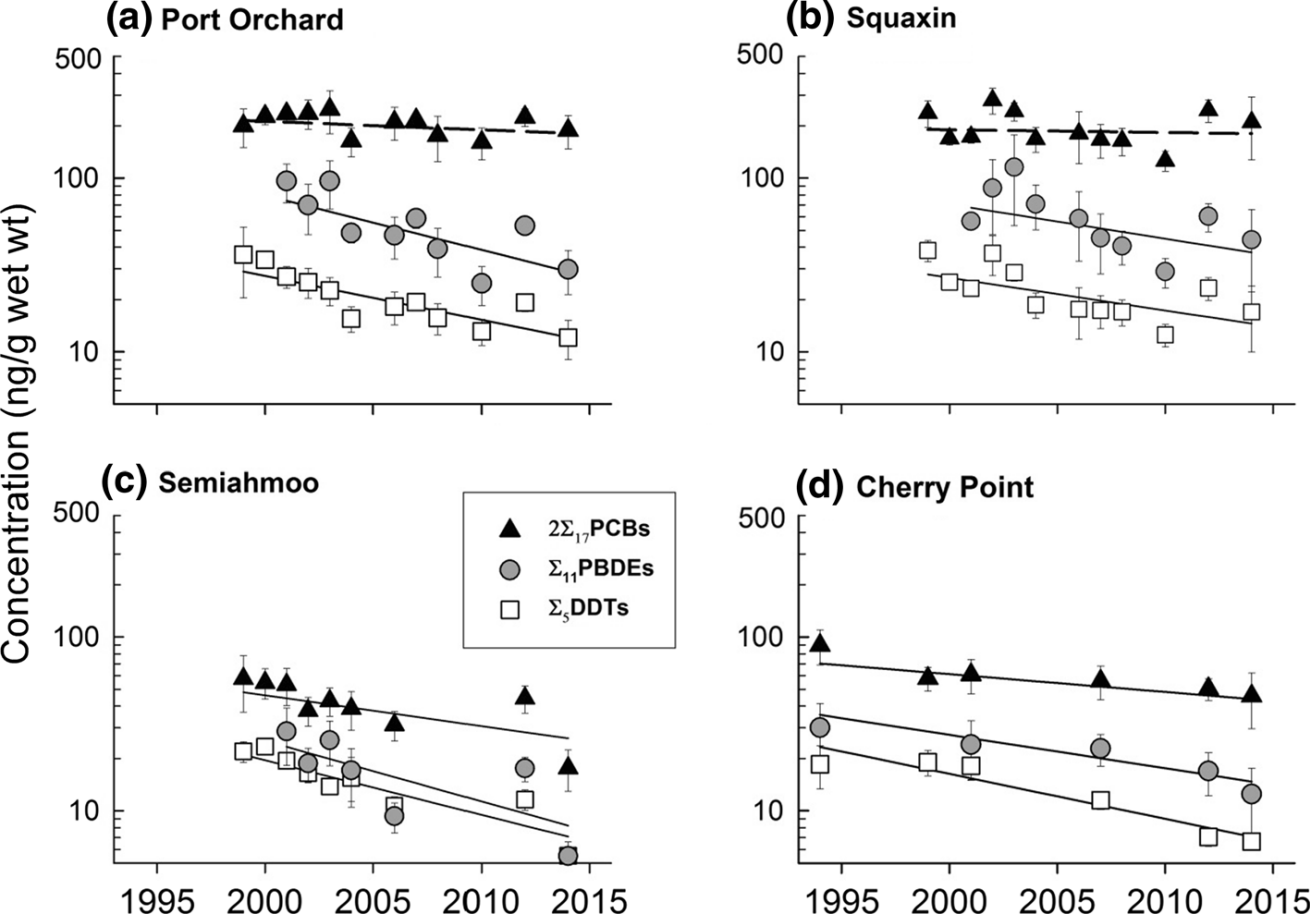
Temporal trend in 2∑_17_PCBs, ∑_11_PBDEs, and ∑_5_DDTs in Pacific herring from four stocks in Puget Sound, WA, sampled from a highly developed basin (Port Orchard stock), moderately developed basin (Squaxin stock) and low-development basin (Semiahmoo and Cherry Point stocks). *Dashed lines* indicate regression models where year was not significant (*p* > 0.05) or censored as trivial (partial *r* ^2^ < 0.10)

Concentrations of 2∑_17_PCBs remained unchanged over the course of the study in herring stocks from Port Orchard (highly developed basin) and Squaxin (moderately developed basin). The year coefficient for 2∑_17_PCBs in Port Orchard herring was censored, because it was both indistinguishable from SRM variability and its partial correlation coefficient was <0.10; hence a 2∑_17_PCBs rate was reported as not significant for that stock (Table 2). Year was not significant for Squaxin in the multiple linear regression (*p* > 0.05). Even though year was not a significant predictor of 2∑_17_PCBs for these two stocks, a predicted (dashed) line was calculated (shown in Fig. 4 for reference). Levels of 2∑_17_PCBs in both herring stocks from the northern, low-development basin (Semiahmoo and Cherry Point stocks) declined significantly, with annual rates of 4.0 and 2.3% (Fig. 4; Table 2). MCL and lipids were either not significant, or weakly to moderately predictive in all stocks, with partial correlation coefficients ranging from 0.02 to 0.44. 2∑_17_PCBs in all predicted lines calculated for Fig. 4 were adjusted for significant covariates.

**Table 2:** Adjusted coefficients of determination (adj  *r* ^2^) for forward stepwise multiple linear regression models and partial *r* ^*2*^ for each significant factor, for models testing the predictive value of year, sex ratio (% of male fish in a composite for English sole only), tissue lipid concentration (%), and fish size (mean composite length in mm) on log-transformed PCBs, PBDEs, and DDTs. Mean composite length (MCL) is the mean standard length in mm of all fish in each composite sample. *n* is the number of composite samples analyzed. *p* indicates the probability for the full regression model. Rate (%) is the back-calculated slope of the year coefficient of the full model. Significant partial *r* ^2^ values (*p* < 0.05) are bolded and were retained in the final model. In any case where either the year coefficient was not significant (ns, *p* > 0.05), or the year coefficient was censored because its partial correlation coefficient was <0.10 (shown in italics), or the rate fell within the 95% confidence interval for the standard reference material % recovery (“Method Performance” section), the rate % was reported as not significant (ns), and the predicted line was dashed in Figs. 3 and 4

	2∑_17_PCBs (ng/g)								∑_11_PBDEs (ng/g)								∑_5_DDTs (ng/g)							
	*n*	Year	Sex Ratio	Lipids (%)	MCL (mm)	adj *r* ^2^	Rate (%)	*p*	*n*	Year	Sex Ratio	Lipids (%)	MCL (mm)	adj *r* ^2^	Rate (%)	*p*	*n*	Year	Sex Ratio	Lipids (%)	MCL (mm)	adj *r* ^2^	Rate (%)	*p*
*Herring* ^a^																								
CP	40	**0.21**	–	ns	ns	0.20	−2.3	0.001	36	**0.41**	–	ns	**0.16**	0.54	−4.3	<0.001	53	**0.83**	–	ns	**0.04**	0.87	−5.8	<0.001
SM	85	**0.34**	–	**0.04**	**0.08**	0.44	−4.0	<0.001	47	**0.37**	–	ns	**0.35**	0.71	−8.1	<0.000	85	**0.75**	–	ns	**0.08**	0.83	−7.0	<0.001
PO	116	***0.03***	–	ns	**0.18**	0.19	ns	<0.001	78	**0.35**	–	ns	**0.28**	0.62	−7.0	<0.001	116	**0.47**	–	ns	**0.12**	0.59	−5.6	<0.001
SQ	115	ns	–	**0.02**	**0.44**	0.45	ns	<0.001	73	**0.11**	–	**0.02**	**0.41**	0.52	−4.5	<0.001	115	**0.24**	–	**0.01**	**0.33**	0.54	−4.2	<0.001
*Sole* ^b^																								
SG	55	ns	**0.1**	ns	ns	ns	ns	0.07	33	ns	ns	ns	**0.56**	0.56	ns	<0.001	55	**0.21**	**0.08**	**0.10**	ns	0.40	−4.2	<0.001
VI	55	ns	ns	ns	ns	na	ns	>0.10	36	**0.17**	**0.08**	**0.10**	ns	0.29	−7.2	0.0033	55	ns	ns	**0.19**	**0.06**	0.23	ns	0.001
EV	55	**0.41**	ns	**0.12**	**0.10**	0.61	7.1	<0.001	34	***0.08***	**0.08**	ns	ns	0.11	ns	0.065	55	ns	ns	**0.13**	**0.11**	0.22	ns	0.001
HC	57	***0.06***	**0.30**	**0.02**	**0.10**	0.44	ns	<0.001	41	***0.05***	**0.09**	**0.31**	ns	0.39	ns	0.0008	57	***0.08***	**0.14**	**0.15**	**0.07**	0.40	ns	<0.001
EH	31	**0.23**	ns	ns	ns	0.21	2.9	0.006	19	ns	ns	**0.22**	**0.19**	0.34	ns	0.014	31	ns	ns	ns	ns	–	ns	0.22
DR	26	ns	**0.20**	ns	ns	0.17	ns	0.021	26	ns	ns	ns	ns	ns	ns	ns	26	**0.29**	**0.09**	ns	ns	0.32	−4.2	0.004
SW	56	**0.20**	ns	ns	**0.08**	0.26	3.6	<0.001	35	**0.11**	ns	**0.06**	ns	0.12	−3.7	0.052	61	ns	**0.08**	ns	**0.12**	0.17	ns	0.003
BR	52	ns	**0.06**	**0.08**	**0.05**	0.23	ns	0.001	37	**0.15**	**0.07**	ns	**0.22**	0.38	−4.0	0.0003	52	**0.16**	ns	**0.05**	ns	0.18	−3.6	0.003
TW	56	**0.16**	**0.28**	**0.12**	ns	0.53	6.2	<0.001	31	ns	**0.22**	**0.13**	**0.15**	0.44	ns	0.0003	51	**0.10**	0.27	**0.12**	ns	0.46	5.2	<0.001
AI	57	***0.04***	**0.19**	**0.05**	ns	0.24	ns	0.001	36	ns	**0.14**	**0.08**	ns	0.22	ns	0.0074	57	***0.06***	ns	**0.08**	ns	0.11	ns	0.018

^a^Herring locations: *CP* Cherry Point; *SM* Semiahmoo; *PO* Port Orchard; *SQ* Squaxin

^b^English sole locations: *SG* Strait of Georgia; *VI*, Vendovi Island; *EV*, Everett; *HC*, Hood Canal; *EH* Eagle Harbor; *DR* Duwamish River; *SW* Seattle waterfront; *BR* Bremerton; *TW* Tacoma City waterway; *AI* Anderson Island

The ∑_11_PBDE levels declined in all herring stocks from all basins, with annual reductions ranging from 4.3 to 8.1%. Fish size (MCL) contributed significantly to explaining PBDE variation with partial *r* ^2^ ranging from 0.16 to 0.41), and lipids were weakly predictive for Squaxin (*r* ^2^ = 0.02, *p* < 0.05) or insignificant (*p* > 0.05) for the other three stocks (Table 2). Final PBDE regression models were moderately predictive, with *r* ^2^ ranging from 0.52 to 0.71; predicted lines were adjusted using grand mean values for significant coefficients (Fig. 4).

As with ∑_11_PBDEs, ∑_5_DDTs declined in all herring stocks, with rates ranging from 5.6% (Port Orchard) in the highly developed basin, 4.2% from Squaxin in the moderately developed basin, to 5.8 and 7.0% in the two stocks from the low-development basin (Cherry Point and Semiahmoo; Fig. 4). Final DDT models were moderately to strongly predictive, with *r* ^2^ ranging from 0.54 to 0.87 (Table 2). Along with year, MCL was a significant partial predictor for all four stocks, albeit most strongly for Squaxin herring, and grand mean values for significant covariates were used to adjust the predicted lines in Fig. 4.

### English Sole Current Status

Concentrations of 2∑_17_PCBs were generally highest in English sole at highly developed sites; in the most current sampling year (2015) arithmetic mean concentrations ranged from 58, 99, and 160 ng/g wet wt (Bremerton, Tacoma City Waterway, and Seattle Waterfront) to 270 ng/g wet wt from the Duwamish River (Table 1; Fig. 3). Fish from moderately developed locations had intermediate concentrations (24–29 ng/g wet wt from Everett and Eagle Harbor) and sole from the low-development locations had the lowest concentrations; 20 ng/g wet wt from Anderson Island, and less than 10 ng/g wet wt from the remaining three.

As with 2∑_17_PCBs, the greatest ∑_11_PBDE concentrations in 2015 occurred in three highly developed locations, although concentrations were relatively low overall: 4.3, 4.5, and 6.0 ng/g wet wt from Duwamish River, Seattle Waterfront, and Tacoma City Waterway (Table 1; Fig. 3). Concentrations of ∑_11_PBDEs in the seven locations with moderate to low development were less than 2 ng/g wet wt. Similar to ∑_11_PBDE, DDTs were low overall, with concentrations ranging from 0.32 to 4.0 ng/g wet wt across all locations. Greatest ∑_5_DDT concentrations occurred at the four highly developed sites, Duwamish River, Seattle Waterfront, Tacoma City Waterway, and Bremerton (4.0–1.3 ng/g wet wt).

Although fish length, percent lipid, and sex ratio covariates (or their interactions) were sometimes significant in the ANCOVA models comparing locations in the current status analysis, they always contributed a minor amount (<3%) in explaining the variability in concentration among the ten sites for any of the POPs. Hence, to simplify the site locations analyses, all geometric means reported in Fig. 3 for English sole POPs were computed without covariate adjustments.

### English Sole Time Trends

**Fig. 5:**
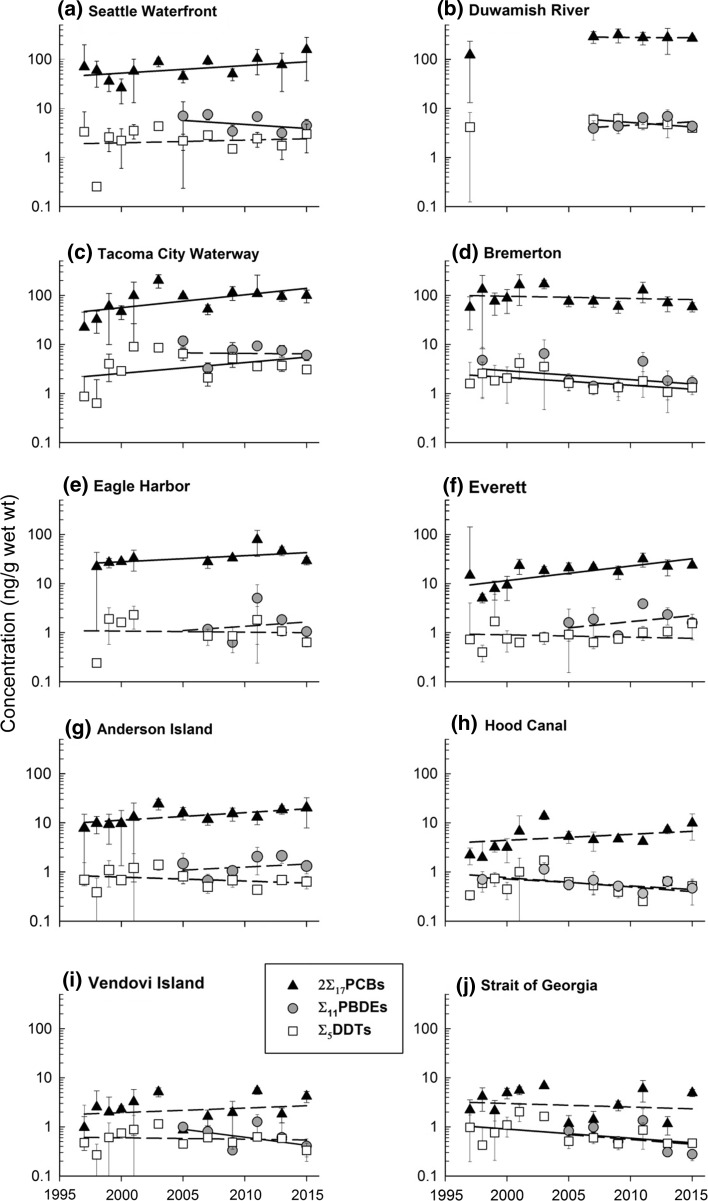
Temporal trend in 2∑_17_PCBs, ∑_11_PBDEs, and ∑_5_DDTs in English sole from ten index sites in Puget Sound. *Dashed lines* indicate regression models where year was not significant (*p* > 0.05) or censored as trivial (partial *r* ^2^ < 0.10)

We did not observe a declining trend in 2∑_17_PCBs from any of the 10 index sites we monitored over the 18-year period from 1997 to 2015 (Fig. 5). Positive, statistically significant (*p* < 0.05) year coefficients indicated increasing PCB concentration at two highly developed sites (Tacoma City Waterway and Seattle Waterfront) and two moderately developed sites (Everett and Eagle Harbor), with annual increases from 2.9% (Eagle Harbor) to 7.1% (Everett; Table 2). Year was weakly to moderately predictive for these sites (partial *r* ^2^ ranging from 0.16 to 0.41; Table 2), and the overall multiple linear regression models for these sites were at best moderately predictive, with final, adjusted *r* ^2^ slightly above 0.50 for only two sites, Tacoma City Waterway, and Everett. English sole from the remaining six index sites exhibited no significant change in PCB concentration over the 19 years. The year coefficient for four of these six sites was not significant (*p* > 0.05), and it was censored as not significant because its partial *r* ^2^ was <0.10 at two sites (Anderson Island and Hood Canal). Sex ratio, lipids, or MCL contributed significantly to the final model for eight sites overall and predicted lines were adjusted using grand mean values of the coefficients for significant covariates (Fig. 5). Predicted lines for sites where there was no significant trend were calculated and are shown in Fig. 5 as dashed, for reference.

Although year was a weak predictor for ∑_11_PBDEs overall in English sole (*r* ^2^ ranged from 0.11 to 0.17), the concentrations of ∑_11_PBDEs with *r* ^2^ > 0.10 declined significantly at two highly developed sites (Bremerton, 4.0% and Seattle Waterfront, 3.7%) and at one low-development site (Vendovi Island, 7.2%; Fig. 5; Table 2). The year coefficient was not significant for five sites (*p* > 0.05) and it was censored as not significant, because its partial correlation coefficient was <0.10 at two sites (Everett and Hood Canal). Fish size, lipids, and sex ratio were weakly to moderately predictive (*r* ^2^ from 0.06 to 0.56) for nine sites, and predicted lines were adjusted using grand mean coefficient values for significant covariates (Fig. 5). Predicted lines for sites where the time trend was not significant were calculated and are shown in Fig. 5 as dashed, for reference.

Concentrations of ∑_5_DDTs declined in English sole from two highly developed sites (Duwamish River, 4.2% and Bremerton, 3.6%) and from one low-development site (Strait of Georgia, 4.2%; Table 2; Fig. 5). One highly developed site (Tacoma City Waterway) exhibited an increase in ∑_5_DDTs, with an annual rate of 5.2%. Year was not significant (*p* > 0.05) or censored as not significant (*r* ^2^ < 0.10) for the remaining six sites (Table 2). As with 2∑_17_PCBs and ∑_11_PBDE, full models for ∑_5_DDTs were weakly to moderately predictive, with *r* ^2^ ranging from 0.11 to 0.46. Predicted lines shown in Fig. 5 were calculated using grand mean values for significant covariates. Predicted lines for sites where the ∑_5_DDTs rate was not significant are shown as dashed, for reference.

## Discussion

### Consistency in Monitoring Data

Monitoring programs are challenged by continually shifting and changing analytical methodologies. Two practices can mitigate loss of consistency when methods change: (1) conducting paired analyses by both old and new methods to generate correction factors, and (2) retaining archived tissues for reanalysis once new methods are developed, validated, and then adopted. Each of these practices requires additional cost for duplicate or reanalysis or raises concerns related to sample quality over time. In this study we employed both practices: PBDEs were always conducted using one methodology, with older archived samples analyzed along with current samples to build a time series. Other long-term monitoring studies have employed this practice, including Ross et al. (2013) who used it to construct the Puget Sound harbor seal time trend line reported earlier herein. Correction factors generated from comparing outdated methods with newer methods appeared to have provided a good tool for correcting biases in our long-term monitoring program. Linear regressions of paired data (old vs. new methods) typically provided excellent fit, with strong coefficients of determination (usually >0.90), and sufficient homoscedasticity to accept significant correlation coefficients as correction factors.

### POPs in Herring

The long-term trend analyses indicate PCB tissue residues declined in both stocks of Pacific herring from the low-development basin, however they remained unchanged in the highly and moderately developed basins. This pattern is consistent with PCB trends in harbor seals, a primary predator of herring, sampled from the south Puget Sound, the moderately developed basin. Although harbor seals exhibited a steep decline in blubber PCBs from the 1980s to 1990s (prior to this study), PCBs remained unchanged or declined only slightly in the most recent years (2003–2009; Ross et al. 2013). A number of studies have demonstrated exponential declines in PCBs in pelagic species in marine and large freshwater ecosystems in the 1980s, with concentrations leveling off over the past decade or two, including herring (Bignert et al. 1998; Miller et al. 2013) and sprat (Szlinder-Richert et al. 2009) from the Baltic Sea, and marine fishes, birds, and mammals from the Canadian Arctic (Braune et al. 2005). Coho and Chinook salmon in the US Great Lakes followed a rapid first order kinetics PCB decline in the 1980s but subsequently leveled off to a non-zero steady state (Stow et al. 1994; Lamon et al. 1999).

PCB concentrations in central and southern Puget Sound herring were considerably higher than levels reported in herring from the most highly developed areas of the southern Baltic Sea sampled during the same time period. Szlinder-Richert et al. (2009) reported mean ICES_7_ PCBs in muscle tissue of Baltic herring ranging from 15 to 27 ng/g wet wt and 360–820 ng/g lipid wt from 1997 to 2006. Because we sampled whole male fish of a relatively fixed size and reported an estimate of total PCBs (2∑_17_PCBs), a direct comparison of PCB wet wt results was inappropriate. Instead, we limited our comparison to concentrations of ICES_7_ PCBs on lipid weight to mitigate the difference in tissue types and PCB summation methods. Whole Pacific herring from central and southern Puget Sound were roughly two times higher than those reported in southern Baltic herring muscle samples, with ICES_7_ PCBs calculated in the Puget Sound herring ranging from 690 to 1800 ng/g lipid weight from a similar time period (1999–2006; Table 1). Southern Baltic herring PCB levels were more similar to concentrations we observed in the two stocks from the Strait of Georgia (least-developed basin), with concentrations ranging from 250 to 900 ng/g lipid. These comparisons were somewhat confounded by differences in fish size and sex; southern Baltic herring from Szlinder-Richert et al. (2009) were larger, with maximum sizes reported as 305 mm compared with mean composite lengths of Pacific herring ranging from 143 to 181 mm. Based on size, Pacific herring in the current study were likely younger than those used in the Baltic study, and so may have experienced a shorter duration of exposure to PCBs. Moreover, the selection of only male herring in the Puget Sound study likely would have increased PCB differences if female fish (represented in the Baltic samples) were depurating POPs during reproduction.

PCB concentrations in Puget Sound herring were within the range reported for two forage fish species, silversides (*Menidia audens*) and topsmelt (*Atherinops affinis*), in San Francisco Bay from 2010 (Greenfield and Allen 2013). These authors reported mean concentrations for sum of 209 PCBs (equivalent to our 2∑_17_PCBs) of 138 ng/g wet wt from “ambient,” randomly selected sites, and 441 ng/g wet wt from sites known to be highly PCB-contaminated. These species were selected by Greenfield and Allen (2013) for similar reasons herring were selected in the present study; herring, topsmelt, and silversides represent small, schooling, midwater prey upon which many higher predators rely and therefore can illustrate an important pathway for POPs like PCBs. Some significant differences between the species prevent an unqualified comparison: the San Francisco silversides and topsmelt were much smaller, roughly half the size of Puget Sound herring, and they exhibited somewhat lower lipids than two of the Puget Sound stocks. Additionally, they likely exhibit somewhat different feeding ecology; topsmelt and silversides were thought to represent local sediment conditions, by feeding on organisms near to the seafloor in shallow waters, whereas herring in Puget Sound likely forage more widely, in much deeper waters, on more pelagic prey.

These Puget Sound and other studies underscore the environmentally recalcitrant characteristics of PCBs, the importance of pelagic biota as a sink for these chemicals, and the role pelagic fishes play as vectors of PCBs to apex predators. Rice et al. (2012) reported Pacific herring as the second most frequently encountered forage fish in a study of Puget Sound surface waters, after juvenile Chinook salmon. These species were encountered in well over 50% of all surface trawls conducted in the same basins represented in the current study. Pacific herring are thought to exert bottom-up ecosystem control in Puget Sound, based on Ecopath/Ecosim modeling (Harvey et al. 2012), and indeed they are one of several midwater fishes that dominate the diet of harbor seals, Puget Sound’s most abundant resident fish-eating marine mammal. Pacific herring, tomcod, hake and salmon were estimated to constitute over 60% of harbor seal diet (Cullon et al. 2005).

O’Neill and West (2009) posited residence time in Puget Sound (i.e., feeding on Puget Sound-resident prey) as a risk factor for PCB accumulation in migratory species that move through this system. Elevated levels of PCBs have been documented in species throughout Puget Sound’s pelagic food web, from plankton (West et al. 2011a) to Puget Sound-resident pelagic fish predators (West et al. 2011b), migratory adult salmon (Cullon et al. 2009; O’Neill and West 2009), marine birds (Good et al. 2014), and marine mammals including resident harbor seals (Ross et al. 2004) and migratory fish-eating killer whales (Ross et al. 2000, Krahn et al. 2007, 2009).

The monitoring program forming the basis of this paper compares POP tissue residues in fish to published critical body residues (CBR; Meador et al. 2011) to predict health impairment. Using this CBR concept, PCB and PBDE concentrations in some herring stocks are cause for concern. Current (2014) PCB concentrations in herring from Port Orchard (3400 ng/g lipid) and Squaxin (4800 ng/g lipid) stocks in this study (Table 1) exceeded a 2400 ng/g health effects threshold determined for juvenile salmon (Meador et al. 2002; the most appropriate CBR for herring from current literature). Because herring sampled in this study were male fish targeted at age 3 years, it is likely that older male fish could have substantially higher POP concentrations (and, conversely, lower levels in younger fish and females of spawning age). Juvenile salmon exposed to PCBs in the range of exposures we observed in Squaxin and Port Orchard herring exhibited a number of health effects including increased enzyme activity, altered thyroid hormones, and increased mortality. Trophic transfer of PCBs via herring also presents a health risk to their predators; 22% of adult Chinook salmon sampled from Puget Sound exceeded the Meador et al. (2002) CBR for PCBs (O’Neill and West 2009). Furthermore, adult Chinook salmon (Cullon et al. 2009; Mongillo et al. 2016) and other pelagic fishes (Cullon et al. 2005; West et al. 2011b) from the Salish Sea (including Puget Sound) were identified as key vectors of PCBs to killer whales and harbor seals, both important apex predators in the ecosystem. Using this exposure pathway, Hickie et al. (2007) predicted PCBs in Southern Resident killer whales feeding in Puget Sound would not fall below a 17-mg total PCBs/kg blubber lipids health-effects threshold concentration until the year 2063. Additionally, the Washington Department of Health (WDOH) has recommended restricting human consumption of Chinook salmon residing in Puget Sound because of PCB contamination in that species (Washington Department of Health 2015).

 Although PCBs are still of significant concern in Puget Sound’s pelagic food web almost 40 years after their production and legal use was banned, decreasing trends of PBDEs and DDTs in all herring stocks point to the success of remediation and control efforts for POPs in Puget Sound. Washington State recommended reductions in PBDE use as early as 2006 (Washington Department of Ecology 2006), and its state legislature restricted further use of PBDEs in 2008. These actions were taken to reduce exposure of humans to PBDEs and to mitigate input of PBDEs to Puget Sound. PBDE reductions notwithstanding, current (2014) tissue levels may still be high enough to impair herring health. Methodological differences in measuring and reporting PBDE concentrations hinder direct interpretation of published PBDE CBRs with the concentrations we reported in Table 2. However, Tomy et al. (2004) and Arkoosh et al. (2017) reported variations in thyroid homeostasis related to dietary doses of PBDEs that produced tissue burdens similar to those we observed in wild herring. In particular, Arkoosh et al. (2017) observed depressed tri-iodothyronine (T3) in plasma of juvenile Chinook salmon with a PBDE body burden of 37 ng/g wet wt (for a mixture of two dominant congeners typically observed in fish samples, PBDE 47 and 99). We reported a similar range of ∑_11_PBDE concentrations; 30 and 44 ng/g wet wt from Squaxin and Port Orchard herring stocks in 2014, and PBDE 47 and 99 were also the most abundant congeners in our samples, accounting for approximately 67% of the ∑_11_PBDE concentration. Tomy et al. (2004) reported depressed thyroxine levels in plasma of lake trout fed “low” and “high” doses of a mixture of 13 PBDEs resulting in maximum lipid-adjusted tissue concentrations of 150 to 1600 ng PBDEs/g lipids (manual summation of concentrations in their Fig. 1) compared with 410–1000 ng/g lipids for the four herring stocks that we reported from Puget Sound. Arkoosh et al. (2010; Fig. 2a) reported increased disease susceptibility in juvenile Chinook salmon dosed with five commonly observed PBDE congeners (47, 99, 100, 153, and 154) resulting in total PBDE concentrations similar to what we observed in our field sampled herring. Assuming that herring respond similarly to Chinook salmon, we would predict some impairment of herring health related to PBDE exposure.

Unlike PCBs and PBDEs, DDT concentrations in all herring were well below a CBR for whole fish of 600 ng/g wet wt (Beckvar et al. 2005); the greatest concentration of ∑_5_DDTs observed in the current study was 39 ng/g wet wt, suggesting DDTs alone posed less risk to herring than the other POPs we measured. However, a tissue residue approach designed to evaluate the toxicity of chemical mixtures (Dyer et al. 2011) for the 3 POP categories we measured may be appropriate for future analyses.

### POPs in English Sole

Whereas Pacific herring (this report) and other pelagic species (West et al. 2011a, b) exhibited pervasive POP contamination of the pelagic food web in highly or moderately developed oceanographic basins (on the order of hundreds of square km) in Puget Sound, English sole illustrated POP conditions at a smaller spatial scale (probably a few square km), related to their proximity to contaminated sediments and limited home range (O’Neill et al. 2007). Results reported for English sole herein are congruent with sediment conditions reported by a companion Puget Sound sediment monitoring program, which measured contaminants in sediments collected near our English sole sampling sites. Long et al. (2005) characterized only approximately 1% of Puget Sound sediments as being significantly impaired by toxic chemicals, including PCBs and DDTs, all of which occurred in close proximity to the same four urban centers where we observed the most contaminated English sole (the bays and shorelines near Seattle, Tacoma, Bremerton, and Everett). English sole exhibited feeding-site fidelity and a small foraging area in an acoustic tagging study (O’Neill et al. 2007), further validating the use of this species as an indicator of more local, bay-scale conditions.

Relatively high PCB concentrations and their increase (or lack of decline) in English sole indicates efforts to control these chemicals in Puget Sound waters have not been effective enough to achieve declining trends. Moreover, mean ICES_7_ PCB levels in English sole from four urban sites (55 ng/g from Seattle Waterfront, Tacoma City Waterway, Duwamish River and Bremerton sites combined) were approximately 12 times higher than the 4.6 ng/g wet wt reported in confamilial European flounder (*Platichthys flesus*) from densely populated and highly urbanized areas in the Baltic Sea in the most recent year when both programs measured these species [Table 1 herein, and Table 2 in Szlinder-Richert et al. (2009)]. Unlike Pacific herring, comparison between Puget Sound and Baltic flatfishes was not confounded by tissue type (skin-off fillets were analyzed for both), so comparison of the original wet wt was appropriate, and only required a correction from the 2∑_17_PCBs to ICES_7_PCBs for the English sole. Based on ICES7 PCBs, the PCB levels in European flounder from urbanized areas of the Baltic Sea Szlinder-Richert et al. 2009) were more similar to PCBs in English sole from the least developed Puget Sound sites (3.8 ng/g wet wt; mean from Strait of Georgia, Vendovi Island, Hood Canal, and Anderson Island).

These observations are consistent with Brown et al. (1998), who documented increasing PCBs in English sole from 1984 to 1990 at two highly developed sites near the Seattle Waterfront and the Tacoma City Waterway sampled in the current study. They also reported no change in PCBs near our low-development Anderson Island site, where we also reported no declining PCB trend. Brown et al. (1998) concluded that redistribution of sediment particles from depositional areas in deeper parts of the contaminated urban bays or continued inputs from existing onshore sources likely accounted for the increase trends they observed in PCB concentration at these highly developed sites. Although it is difficult to track all in-water activities in the urban waterways where English sole were collected for this study, many of them experienced significant anthropogenic sediment-disturbing activities throughout our 19-year monitoring period, including sediment removal via dredging for navigation or for contaminant remediation. Other factors that may have prevented PCB declines in English sole include: (1) recirculation of persistent congeners in the benthic food web; (2) new PCB inputs that have not been adequately controlled; and (3) increasing wastewater and stormwater conveyance of PCBs related to increases in human population in the region that may offset source control efforts.

The ∑_11_PBDEs and ∑_5_DDTs declined or remained unchanged in all English sole with one exception, the 5.2% annual increase of ∑_5_DDTs in English sole from the Tacoma City Waterway. The levels of ∑_11_PBDEs and ∑_5_DDTs at many English sole sites were heading in a desirable direction (declining) and all concentrations were low (especially compared with 2∑_17_PCBs), with values that varied narrowly, near the limit of quantitation for many of the analytes used in the summations. In these cases, trends should be interpreted with caution because of the possibility of true trends being obscured by method performance near the limits of quantitation. In addition, recovery of DDT analytes in standard reference materials was particularly problematic because of interference with unknown chemicals, and inconsistent performance through time that was difficult to quantify. These cautions notwithstanding, ∑_5_DDTs in English sole from the Tacoma City Waterway is particularly notable, because it showed a relatively strong increasing trend. The Tacoma City Waterway index site is located near the mouth of the Puyallup River, which receives runoff from agricultural, residential, and urbanized lands. Johnson et al. (1988) noted recalcitrant DDTs from agricultural soils can be a significant source of DDTs in Washington rivers as a result of erosion, which may explain the source of DDTs at the Tacoma site. This site also has experienced a number of dredging and cleanup operations, as well as removal of contaminated shoreline soils, which may have exposed otherwise sequestered DDTs.

Similar to San Francisco Bay marine fish (Davis et al. 2007), a primary concern regarding PCBs in English sole is protection of human health from contaminated seafood. PCB concentrations in English sole were high enough to generate advice restricting consumption from five of the ten Puget Sound index sites in the current report; Tacoma City Waterway, Duwamish River, Eagle Harbor, Seattle Waterfront, and Bremerton (Washington Department of Health 2015). Furthermore, PCBs in English sole from these five sites, plus those from Anderson Island and Everett exceeded a human health screening value (10 ng/g wet wt) for marine fish in San Francisco Bay (Davis et al. 2007). PBDEs (0.28–6.0 ng/g wet wt) and DDTs (0.30–3.1 ng/g wet wt) in English sole were low relative to fish-health CBRs; that is, near to PBDE concentrations reported for experimental controls in Arkoosh et al. (2010) and more than 100 times lower than the Beckvar et al. (2005) DDT threshold of 600 ng/g wet wt.

### Challenges to Reducing POPs

Evaluation of best management practices to mitigate POPs in the marine environment requires a full understanding of not only the sources of POPs to the system but also the fate and transport of POPs in the system (Arnot and Gobas 2004; Osterberg and Pelletier 2015). The POPs selected for tracking in this long-term monitoring program cover a wide range of hydrophobicity and half-lives, with most considered environmentally recalcitrant (Johnson et al. 2014). For example, Fisk et al. (1998) reported biomagnification factors (BMF) for the 16 PCB congeners we quantitated ranging from 1 to 16, and half-lives of 44–224 days, with slow or no metabolism of this group in fish (for a low dose scenario). Similarly, Tomy et al. (2004) reported BMFs for PBDEs in lake trout ranging from 2.1 to 18, and half-lives of 38–210 days, although accurate assessment of PBDE BMFs is hampered by biotransformation of highly brominated (e.g., deca-molecules) to lower brominated forms within fish. Although POPs can degrade in the environment by photolysis (e.g., PBDEs; Fang et al. 2008) the more likely degradation mechanisms for POPs in biota are biological, because light may not penetrate solid matrices, such as sediments and tissues (Johnson et al. 2014). Metabolic breakdown of PCBs in marine sediments can occur by bacteria (Papale et al. 2016; Matturro et al. 2016); however, the extent of this degradation capacity is unknown.

POPs in our benthic indicator (English sole) clearly reflected sediment conditions where they foraged; however, POPs may enter pelagic food webs via more complex mechanisms. Others have shown POP uptake into pelagic food webs directly from surface waters via ad/absorption by bacteria, other microplankton, and phytoplankton (Larsson et al. 2000; Hudson et al. 2005). Sinking POP-laden particles that may otherwise reach the seafloor in shallow systems can be intercepted by vertically migrating zooplankton, such as krill (*Euphausia* spp; Dilling et al. 1998) in deeper systems. These species are known to concentrate and feed on sinking particulates at the density gradient, where the sinking rate of particles is slowed (Dilling and Alldredge 2000) and which is a characteristic of deeper, vertically stratified systems, such as Puget Sound. The disparity in POP status and trends we observed between herring and English sole probably reflects these processes, and a deeper investigation of these processes may better inform the development of best management practices to mitigate POPs in systems like Puget Sound. Reduction of POPs in English sole may achieved most directly by reducing POPs in sediments from highly developed embayments. POP reductions in the pelagic food web may be more complicated and more responsive to reducing POPs in the water column, whether originating from stormwater or nonpoint sources, point-source inputs, sediment resuspension, atmospheric transport, or trophic recirculation within the pelagic food web.

## Conclusions

Relatively high and static levels of PCBs in Pacific herring from the moderately and highly developed Puget Sound basins (this study), as well as from resident pelagic fishes in other large coastal estuaries, such as San Francisco Bay (Davis et al. 2007) and the Baltic Sea (Szlinder-Richert et al. 2009) underscore the persistence of PCBs in the pelagic food webs of nearshore, relatively enclosed marine ecosystems. Remediation efforts to date seem to have reduced PCBs from high levels in the 1970s and 1980s; however, levels of significant concern continue. PCBs in Puget Sound’s benthic indicator species appear to be increasing in several urbanized areas, and not decreasing in any sampled location. Particular challenges identified by these studies may be to develop methods for reducing new PCB sources to the pelagic food web and to understand the causes for PCB increases in benthic species from urbanized locations. Puget Sound’s pelagic prey base continues to be a hot-spot of PCBs in the northeastern Pacific Ocean region, resulting in long-term contamination of apex predators and other species important to healthy ecosystem function and to human use. Recovery of at least two species listed for protection and recovery by the U.S. Endangered Species Act and Canadian Species at Risk Act, southern resident killer whales (Krahn et al. 2002) and Chinook salmon (Myers et al. 1998) may be significantly hindered by their exposure to PCBs from their prey base in Puget Sound (Hickie et al. 2007; O’Neill and West 2009; Mongillo et al. 2016). PBDEs and DDTs appear to be on the decline in both benthic and pelagic species, except for a few notable exceptions, suggesting that efforts to reduce or remove these chemicals have been largely successful. Continued tracking of POPs in benthic species, such as English sole, will continue to provide a measure of efficacy of more local cleanup or remediation efforts.

## References

[CR3] Arkoosh MR, Boylen D, Dietrich J, Anulacion BF, Ylitalo G, Bravo CF, Johnson LL, Loge FJ, Collier TK (2010). Disease susceptibility of salmon exposed to polybrominated diphenyl ethers (PBDEs). Aquatic Toxicol.

[CR4] Arkoosh MR, Van Gaest AL, Strickland SA, Hutchinson GP, Krupkin AB, Dietrich JP (2017). Alteration of thyroid hormone concentrations in juvenile chinook salmon (*Oncorhynchus tshawytscha*) exposed to polybrominated diphenyl ethers, BDE-47 and BDE-99. Chemosphere.

[CR5] Arnot JA, Gobas FAPC (2004). A food web bioaccumulation model for organic chemicals in aquatic ecosystems. Environ Toxicol Chem.

[CR6] Beckvar N, Dillon TM, Read LB (2005). Approaches for linking whole-body fish tissue residues of mercury or DDT to biological effects thresholds. Environ Toxicol Chem.

[CR7] Bignert A, Olsson M, Persson W, Jensen S, Zakrisson S, Litzen K, Eriksson U, Haggberg L, Alsberg T (1998). Temporal trends of organochlorines in Northern Europe, 1967–1995. Relation to global fractionation, leakage from sediments and international measures. Environ Poll.

[CR8] Braune BM, Outridge PM, Fisk AT, Muir DCG, Helm PA, Hobbs K, Hoekstra PF, Kuzyk ZA, Kwan M, Letcher RJ, Lockhart WL, Norstrom RJ, Stern GA, Stirling I (2005). Persistent organic pollutants and mercury in marine biota of the Canadian Arctic: an overview of spatial and temporal trends. Sci Total Environ.

[CR9] Brown DW, McCain BB, Horness BH, Sloan CA, Tilbury KL, Pierce SM, Burrows DG, Chan S-L, Landahl JT, Krahn MM (1998). Status, correlations, and temporal trends of chemcial contaminants in fish and sediment from selcted sites on the Pacific Coast of the USA. Mar Pollut Bull.

[CR10] Burns R (1985) The shape and form of Puget Sound. University of Washington, Washington Sea Grant Publication, University of Washington Press, Seattle, p 100

[CR11] Cullon DL, Jeffries SJ, Ross PS (2005). Persistent organic pollutants in the diet of harbor seals (*Phoca vitulina*) inhabiting Puget Sound, Washington (USA), and the Strait of Georgia, British Columbia (Canada): a food basket approach. Environ Toxicol Chem.

[CR12] Cullon DL, Yunker MB, Alleyne C, Dangerfield NJ, O’Neill S, Whiticar MJ, Ross PS (2009). Persistent organic pollutants in Chinook salmon (*Oncorhynchus tshawytscha*): implications for resident killer whales of British Columbia and adjacent waters. Environ Toxicol Chem.

[CR13] Davis JA, Hetzel F, Oram JJ, McKee LJ (2007). Polychlorinated biphenyls (PCBs) in San Francisco Bay. Environ Res.

[CR14] Day DE (1976). Homing behavior and population stratification in central Puget Sound English sole (*Parophrys vetulus*). J Fish Res Bd Canada.

[CR15] Dilling L, Alldredge AL (2000). Fragmentation of marine snow by swimming macrozooplankton: a new process impacting carbon cycling in the sea. Deep Sea Res Part I.

[CR16] Dilling L, Wilson J, Steinberg D, Alldredge A (1998). Feeding by the euphausiid *Euphausia pacifica* and the copepod *Calanus pacificus* on marine snow. Mar Ecol Progress Ser.

[CR17] Dodder NG, Maruya KA, Ferguson PL, Grace R, Klosterhaus S, La GuardiaMJ, Lauenstein GG, Ramirez J (2013). Occurrence of contaminants of emerging concern in mussels (*Mytilus* spp.) along the California coast and the influence of land use, storm water discharge, and treated wastewater effluent. Mar Pollut Bull.

[CR18] Dyer S, Warne SJ, Meyer JS, Leslie HA HA, Escher Bl (2011). Tissue residue approach for chemical mixtures. Integr Environ Assess Manag.

[CR19] Fang L, Huang J, Yu G, Wang L (2008). Photochemical degradation of six polybrominated diphenyl ether congeners under ultraviolet irradiation in hexane. Chemosphere.

[CR20] Fisk AT, Norstrom RJ, Cymbalisty CD, Muir DCG (1998). Dietary accumulation and depuration of hydrophobic organochlorines: bioaccumulation parameters and their relationship with the octanol/water partition coefficient. Environ Toxicol Chem.

[CR21] Fry J, Xian G, Jin S (2011). Completion of the 2006 national land cover database for the conterminous United States. Photogramm Eng Remote Sens.

[CR22] Good TP, Pearson SF, Hodum P, Boyd D, Anulacion BF, Ylitalo GM (2014). Persistent organic pollutants in forage fish prey of rhinoceros auklets breeding in Puget Sound and the northern California Current. Mar Pollut Bull.

[CR23] Greenfield BK, Allen RM (2013). Polychlorinated biphenyl spatial patterns in San Francisco Bay forage fish. Chemosphere.

[CR1] Harrison PJ, Mackas DL, Frost BW, MacDonald RW, Crecelius EA (1994) An assessment of nutrients, plankton, and some pollutants in the water column of Juan de Fuca Strait, Strait of Georgia and Puget Sound, and their transboundary transport. Review of the marine environment and biota of Strait of Georgia, Puget Sound, and Juan de Fuca Strait: proceedings of the BC/Washington symposium on the marine environment, Jan 13 & 14, 1994. Can Tech Rep Fish Aquat Sci Report No. 1948

[CR24] Harvey CJ, Williams GD, Levin PS (2012). Food web structure and trophic control in central Puget Sound. Estuaries Coasts.

[CR25] Hickie BE, Ross PS, MacDonald RW, Ford JKB (2007). Killer whales (*Orcinus orca*) face protracted health risks associated with lifetime exposure to PCBs. Environ Sci Technol.

[CR26] Hudson MJ, Swackhamer DL, Cotner JB (2005). Effect of microbes on contaminant transfer in the lake superior food web. Environ Sci Technol.

[CR27] International Council for the Exploration of the Sea (1987) Report of the ICES advisory committee on marine pollution, 1986, International Council for the Exploration of the Sea. Issue 142 of Cooperative Research Report

[CR71] Jensen S, Johnels AG, Olsson M, Otterlind G (1969) DDT and PCB in marine animals from Swedish waters. Nature 224(5216):247–250 10.1038/224247a0 5388040

[CR28] Johnson A, Norton D, Yake B (1988). Persistence of DDT in the Yakima River drainage, Washington. Arch Environ Contam Toxicol.

[CR29] Johnson LL, Anulacion BF, Arkoosh MR, Burrows DG, DaSilva DAM, Dietrich JP, Tierney KB, Farrell AP, Brauner CJ (2014). Effects of legacy peristent organic pollutants (pops) in fish—current and future challenges. Organic chemical toxicology of fishes: fish physiology.

[CR30] Jones KC, de Voogt P (1999). Persistent organic pollutants (POPs): state of the science. Environ Poll.

[CR31] Krahn MM, Ylitalo GM, Buzitis J, Sloan CA, Boyd DT, Chan S-L, Varanasi U (1994). Screening for planar chlorobiphenyl congeners in tissues of marine biota by high performance liquid chromatography with photodiode array detection. Chemosphere.

[CR32] Krahn MM, Wade PR, Kalinowski S, Dalheim ME, Taylor BL et al (2002) Status review of Southern resident killer whales (*Orcinus orca*) under the endangered species act. NOAA Technical Memorandum NMFS-NWFSC-54

[CR33] Krahn MM, Hanson MB, Baird RW, Boyer RH, Burrows DG, Emmons CK, Ford JK, Jones LL, Noren DP, Ross PS, Schorr GS (2007). Persistent organic pollutants and stable isotopes in biopsy samples (2004/2006) from Southern Resident killer whales. Mar Pollut Bull.

[CR34] Krahn MM, Hanson MB, Schorr GS, Emmons CK, Burrows DG, Bolton JL, Baird RW, Ylitalo GM (2009). Effects of age, sex and reproductive status on persistent organic pollutant concentrations in “Southern Resident” killer whales. Mar Pollut Bull.

[CR35] Lamon ECI, Carpenter SR, Stow CA (1999). Rates of decrease of polychlorinated biphenyl concentrations in five species of Lake Michigan salmonids. Can J Fish Aquat Sci.

[CR36] Lanksbury J, Carey A, Niewolny L, West J (2013). Mussel watch pilot expansion 2012/2013: a study of toxic contaminants in blue mussels (*Mytilus trossulus*) from puget sound washington, USA.

[CR37] Larsson P, Andersson A, Broman D, Nordbäck J, Lundberg E (2000). Persistent organic pollutants (POPs) in pelagic systems. Ambio.

[CR38] Lauenstein GG, Cantillo AY (1993) Sampling and analytical methods of the National Status and Trends Program National Benthic Surveillance and Mussel Watch projects. 1984–1992. Silver Spring, MD, National Oceanic and Atmospheric Administration Technical Memorandum NOS ORCA 71

[CR39] Long ER, Dutch M, Aasen S, Welch K, Hameedi MJ (2005). Spatial extent of degraded sediment quality in Puget Sound (Washington State, U.S.A.) based upon measures of the sediment quality triad. Environ Monit Assess.

[CR40] Matturro B, Ubaldi C, Rossetti S (2016). Microbiome dynamics of a polychlorobiphenyl (PCB) historically contaminated marine sediment under conditions promoting reductive dechlorination. Front Microbiol.

[CR41] Meador JP, Collier TK, Stein JE (2002). Use of tissue and sediment-based threshold concentrations of polychlorinated biphenyls (PCBs) to protect juvenile salmonids listed under the US Endangered Species Act. Aquat Conserv.

[CR42] Meador JP, Adams WJ, Escher BI, McCarty LS, McElroy AE, Sappington KG (2011). The tissue residue approach for toxicity assessment: findings and critical reviews from a society of environmental toxicology and chemistry Pellston Workshop. Integr Environ Assess Manag.

[CR43] Miller A, Hedman JE, Nyberg E, Haglund P, Cousins IT, Wiberg K, Bignert A (2013). Temporal trends in dioxins (polychlorinated dibenzo-p-dioxin and dibenzofurans) and dioxin-like polychlorinated biphenyls in Baltic herring (*Clupea harengus*). Mar Pollut Bull.

[CR44] Mongillo TM, Ylitalo GM, Rhodes LD, O’Neill SM, Noren DP, Hanson MB (2016) Exposure to a mixture of toxic chemicals: implications for the health of endangered Southern Resident killer whales. U.S. Dept. Commer, NOAA Tech. Memo. NMFS-NWFSC-135. doi:10.7289/V5/TM-NWFSC-135

[CR45] Moore SK, Mantua NJ, Newton JA, Kawase M, Warner MA, Kellogg JP (2008). A descriptive analysis of temporal and spatial patterns of variability in Puget Sound oceanographic properties. Estuar Coast Shelf Sci.

[CR46] Moser ML, Myers MS, West JE, O’Neill SM, Burke BJ (2013). English sole spawning migration and evidence for feeding site fidelity in Puget Sound, USA, with implications for contaminant exposure. Northwest Sci.

[CR2] Myers JM, Kope RG, Bryant GJ, Teel D, Lierheimeret LJ et al (1998) Status review of chinook salmon from Washington, Idaho, Oregon, and California. US Department of Commerce NOAA Technical Memorandum NMFS-NWFSC-35, Seattle, Washington

[CR74] O’Neill SM, Moser ML, Myers MS, Quinnell SR, West JE (2007) Acoustic telemetry reveals daily movement patterns and annual homing migration to foraging habitats by English sole: application to management of contaminated sediments. Proceedings of the 2007 Puget Sound Georgia Basin Research Conference. http://depts.washington.edu/uwconf/2007psgb/2007proceedings/papers/p12_oneil.pdf

[CR47] O’Neill SM, West JE (2009). Marine distribution, life history traits, and the accumulation of polychlorinated biphenyls in Chinook salmon from Puget Sound, Washington. Trans Am Fish Soc.

[CR48] Osterberg DJ, Pelletier G (2015) Puget sound regional toxics model: evaluation of PCBs, PBDEs, PAHs, copper, lead and zinc. Washington Department of Ecology Report 15-03-025

[CR49] Papale M, Giannarelli S, Francesconi S, Di Marco G, Mikkonen A et al (2016) Enrichment, isolation and biodegradation potential of psychrotolerant polychlorinated-biphenyl degrading bacteria from the Kongsfjorden (Svalbard Islands, high arctic Norway). Mar Poll Bull 114(2):849–859 10.1016/j.marpolbul.2016.11.011 27855955

[CR50] Puget Sound Partnership (2016) Puget Sound vital signs: toxics in fish. http://www.psp.wa.gov/vitalsigns/toxics_in_fish.php

[CR51] Rice CA, Duda JJ, Greene CM, Karr JR (2012). Geographic patterns of fishes and jellyfish in Puget Sound surface waters. Mar Coastal Fish.

[CR75] Ross PS (2006) Fireproof killer whales (*Orcinus orca*): flame-retardant chemicals and the conservation imperative in the charismatic icon of British Columbia, Canada. Can J Fish Aquat Sci 63(1):224–234

[CR53] Ross PS, Ellis GM, Ikonomou MG, Barrett-Lennard LG, Addison RF (2000). High PCB concentrations in free-ranging Pacific killer whales, *Orcinus orca*: effects of age, sex and dietary preference. Mar Pollut Bull.

[CR54] Ross PS, Jeffries SJ, Yunker MB, Addison RF, Ikonomou MG, Calambokidis JC (2004). Harbor seals (*Phoca vitulina*) in British Columbia, Canada, and Washington State, USA reveal a combination of local and global polychlorinated biphenyl, dioxin, and furan signals. Environ Toxicol Chem.

[CR76] Ross PS, Couillard CM, Ikonomou MG, Johannessen SC, Lebeuf M, Macdonald RW, Tomy GT (2009) Large and growing environmental reservoirs of Deca-BDE present an emerging health risk for fish and marine mammals. Mar Pollut Bull 58(1):7–10 10.1016/j.marpolbul.2008.09.002 18929373

[CR56] Ross PS, Noël M, Lambourn D, Dangerfield N, Calambokidis J, Jeffries S (2013). Declining concentrations of persistent PCBs, PBDEs, PCDEs, and PCNs in harbor seals (*Phoca vitulina*) from the Salish Sea. Prog Oceanogr.

[CR57] Sloan CA, Brown DW, Pearce RW, Boyer RH, Bolton JL, Burrows DG, Herman DP, Krahn MM (2004) Extraction, cleanup, and gas chromatography/mass spectrometry analysis of sediments and tissues for organic contaminants. NOAA Technical Memorandum NMFS-NWFSC-59

[CR73] Sloan CA, Brown DW, Pearce RW, Boyer RH, Bolton JL, Burrows DG, Herman DP, Krahn MM (2005) Determining aromatic hydrocarbons and chlorinated hydrocarbons in sediments and tissues using accelerated solvent extraction and gas chromatography/mass spectrometry. In: Ostrander GK (ed) Aquatic toxicology, vol 2. CRC Press, Boca Raton, FL, pp 631–651

[CR58] Sloan CA, Anulacion BF, Baugh KA, Bolton JL, Boyd D, Boyer RH, Burrows DG, Herman DP, Pearce RW, Ylitalo GM (2014) Northwest Fisheries Science Center’s analyses of tissue, sediment, and water samples for organic contaminants by gas chromatography/mass spectrometry and analyses of tissue for lipid classes by thin layer chromatography/flame ionization detection. NOAA Technical Memorandum NMFS-NWFSC-125

[CR59] Stow CA, Carpenter SR, Amrhein JF (1994). PCB concentration trends in Lake Michigan coho (*Oncorhynchus kisutch*) and chinook salmon (*O. tshawytscha*). Can J Fish Aquat Sci.

[CR60] SYSTAT (2007). SYSTAT 12.

[CR61] Szlinder-Richert J, Barska I, Mazerski J, Usydus Z (2009). PCBs in fish from the southern Baltic Sea: levels, bioaccumulation features, and temporal trends during the period from 1997 to 2006. Mar Pollut Bull.

[CR62] Thomson RE (1994) Physical oceanography of the Strait of Georgia-Puget Sound-Juan de Fuca Strait system. Review of the marine environment and biota of Strait of Georgia, Puget Sound, and Juan de Fuca Strait. In: Proceedings of the BC/Washington symposium on the marine environment, Jan 13, 14, 1994. Can Tech Rep Fish Aquat Sci Report No. 1948

[CR63] Tierney KB, Kennedy JC, Gobas F, Gledhill M, Sekela M, Tierney KB, Farrell AP, Brauner CJ (2014). Organic contaminants and fish. Organic chemical toxicology of fishes: Fish physiology.

[CR64] Tomy GT, Palace VP, Halldorson T, Braekevelt E, Danell R, Wautier K (2004). Bioaccumulation, biotransformation, and biochemical effects of brominated diphenyl ethers in juvenile lake trout (*Salvelinus namaycush*). Environ Sci Technol.

[CR72] Washington Department of Ecology (2006) Washington State polybrominated diphenyl ether (PBDE) Chemical Action Plan: Final Plan. Washington Department of Ecology Report 05-07-048. p 328. http://www.ecy.wa.gov/biblio/0507048.html

[CR65] Washington Department of Health (2015) Puget Sound fish consumption advice. Washington Department of Health, Division of Environmental Health, Office of Environmental Health Assessments. Olympia, Washington. http://www.doh.wa.gov/Portals/1/Documents/Pubs/334-098.pdf

[CR66] West JE, O’Neill SM, Ylitalo GM (2008). Spatial extent, magnitude, and patterns of persistent organochlorine pollutants in Pacific herring (*Clupea pallasi*) populations in the Puget Sound (USA) and Strait of Georgia (Canada). Sci Total Environ.

[CR67] West JE, Lanksbury J, O’Neill SM (2011a) Persistent organic pollutants in marine plankton from Puget Sound, Washington Department of Ecology report no. 11-10-002

[CR68] West JE, Lanksbury J, O’Neill SM, Marshall A (2011b) Persistent, bioaccumulative and toxic contaminants in pelagic marine fish species from Puget Sound. Washington Department of Ecology report no. 11-10-003

[CR69] Wickham JD, Stehman SV, Gass L, Dewitz J, Fry JA, Wade TG (2013). Accuracy assessment of NLCD 2006 land cover and impervious surface. Remote Sens Environ.

[CR70] Ylitalo GM, Buzitis J, Krahn MM (1999). Analyses of tissues of eight marine species from Atlantic and Pacific coasts for dioxin-like chlorobiphenyls (CBs) and total CBs. Arch Environ Contam Toxicol.

